# Identification of Dihydroorotate Dehydrogenase Inhibitors Using the Cell Painting Assay

**DOI:** 10.1002/cbic.202200475

**Published:** 2022-10-13

**Authors:** Beate Schölermann, Jana Bonowski, Michael Grigalunas, Annina Burhop, Yusheng Xie, Joseph G. F. Hoock, Jie Liu, Mark Dow, Adam Nelson, Christine Nowak, Axel Pahl, Sonja Sievers, Slava Ziegler

**Affiliations:** ^1^ Max Planck Institute of Molecular Physiology Department of Chemical Biology 44227 Dortmund Germany; ^2^ School of Chemistry and Astbury Centre for Structural Molecular Biology University of Leeds LS2 9JT Leeds UK; ^3^ Compound Management and Screening Center 44227 Dortmund Germany

**Keywords:** enzymes, inhibitors, morphological fingerprint, mode of action, nucleotides, targets

## Abstract

Profiling approaches have been increasingly employed for the characterization of disease‐relevant phenotypes or compound perturbation as they provide a broad, unbiased view on impaired cellular states. We report that morphological profiling using the cell painting assay (CPA) can detect modulators of *de novo* pyrimidine biosynthesis and of dihydroorotate dehydrogenase (DHODH) in particular. The CPA can differentiate between impairment of pyrimidine and folate metabolism, which both affect cellular nucleotide pools. The identified morphological signature is shared by inhibitors of DHODH and the functionally tightly coupled complex III of the mitochondrial respiratory chain as well as by UMP synthase, which is downstream of DHODH. The CPA appears to be particularly suited for the detection of DHODH inhibitors at the site of their action in cells. As DHODH is a validated therapeutic target, the CPA will enable unbiased identification of DHODH inhibitors and inhibitors of *de novo* pyrimidine biosynthesis for biological research and drug discovery.

## Graphical abstract

**A morphological fingerprint** has been identified for the suppression of *de novo* pyrimidine biosynthesis which is shared by inhibitors of enzymes that are involved in pyrimidine synthesis such as DHODH and inhibitors of complex III. This bioactivity cluster can be employed to identify further modulators of these targets.

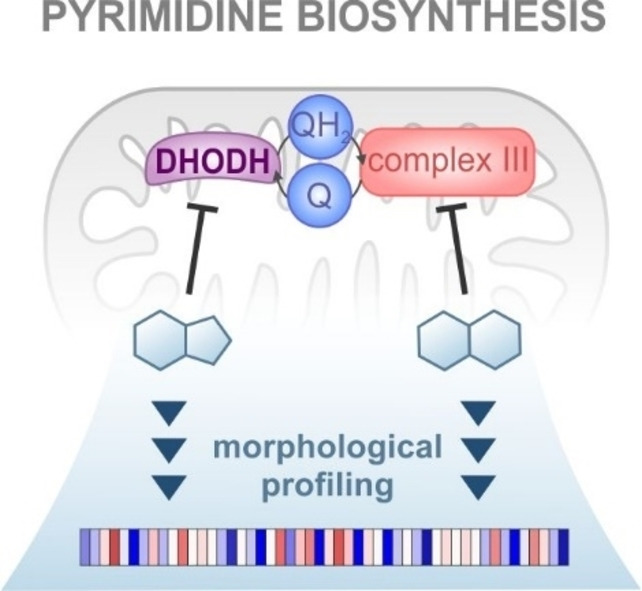

## Introduction

The identification of biologically active compounds is prerequisite for the development of chemical tool compounds to dissect biology or of drugs for therapeutic application. Bioactive small molecules are identified using target‐ or cell‐based (phenotypic) assays. Phenotypic assays monitor modulation of a process of interest at the site of action, i. e., in cells or even organisms, while profiling approaches detect biological activity in a less biased manner, e. g., by recording changes in the expression of thousands of genes, proteins or morphological features. Whereas the throughput of transcriptomics and proteomics has only recently been increased,^[1]^ morphological profiling has been designed from the onset to collect morphological fingerprints in a medium throughput manner. Compounds with similar fingerprints are expected to address the same target or to share the same mode of action (MoA). Thus far, morphological profiling in general and the Cell Painting assay (CPA) in particular has detected modulation of various targets such as BET, HDACs, HSP90, kinases and tubulin or processes like DNA synthesis, protein synthesis, lysosomotropism/cholesterol homeostasis and uncoupling of the mitochondrial proton gradient.^[2]^ There is a high demand to define further reliable CPA bioactivity clusters to cover a broad range of bioactivities that will be used for the generation of target or MoA hypotheses.

Nucleotides are the building blocks of DNA and RNA and are essential for various vital cellular processes such as DNA synthesis and proliferation, transcription, signaling and metabolism.^[3]^ Nucleotide analogs are used as anti‐cancer drugs, however, they do not affect only cancer cells but also normal cells. Deregulation of nucleotide metabolism is linked to several diseases such as cancer and viral infections.^[4]^ Pyrimidine biosynthesis supplies cancer cells with sufficient amounts of nucleotides and sustains membrane biogenesis, cell signaling and metabolism^[5]^ and is an established target for the treatment of autoimmune and viral diseases and cancer.

Here we report the identification of a characteristic morphological fingerprint for the inhibition of dihydroorotate dehydrogenase (DHODH), a rate‐limiting enzyme in pyrimidine biosynthesis, using the CPA. This fingerprint enables mapping of modulators of *de novo* pyrimidine biosynthesis in general and particularly of DHODH. Fingerprint similarity to the DHODH inhibitor brequinar revealed three novel scaffolds for targeting DHODH. Furthermore, impairment of uridine 5’‐monophospate (UMP) synthase or mitochondrial complex III results in fingerprints that are biosimilar to brequinar. Thus, different targets related to pyrimidine biosynthesis can be predicted using CPA. DHODH is a validated target for the treatment of autoimmune diseases, like relapsing multiple sclerosis and rheumatoid arthritis,^[6]^ and DHODH inhibitors promote differentiation of acute myeloid leukemic cells thereby reducing the level of leukemia‐initiating cells.^[7]^ Moreover, DHODH inhibitors are promising agents for the treatment of various viral diseases including SARS‐CoV‐2.^[8]^ The CPA identifies DHODH modulation at the site of its action, i. e., in the mitochondria, and can detect DHODH inhibitors that are only weakly active or even inactive in an *in vitro* DHODH assay. Therefore, this morphological profiling approach will facilitate the discovery of DHODH inhibitors and may spur the development of DHODH‐based therapies.

## Results and Discussion

**Figure 1:**
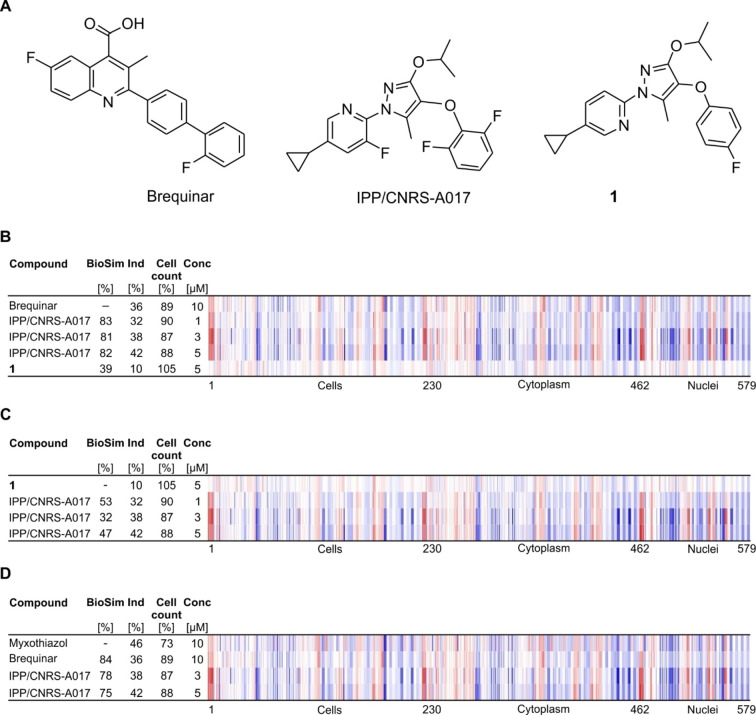
Similarity of DHODH inhibitors in the Cell Painting assay. (A) Structures of brequinar, IPP/CNRS‐A017 (IPP/CNRS) and compound **1**, a much less active derivative of IPP/CNRS‐A017. (B) Fingerprint comparison for brequinar (10 μM), IPP/CNRS‐A017 (1, 3 and 5 μM) and compound **1** (5 μM). (C) Similarity of compound **1** to IPP/CNRS‐A017. (D) Similarity of brequinar and IPP/CNRS‐A017 to the complex III inhibitor myxothiazol. The top line fingerprint is set as a reference fingerprint (100 % biological similarity, BioSim) to which the following fingerprints are compared. Values were normalized to the DMSO control. Blue color: decreased feature, red color: increased feature. The set of 579 features is divided in features related to the cell (1–229), cytoplasm (230–461) and nuclei (462–579). BioSim: biosimilarity, Ind: induction, Conc: concentration.

For reference compounds, i. e., compounds with known target or MoA, morphological fingerprints often do not correlate with their annotated activity but are rather related to an off‐target.^[9]^ Therefore, the definition of bioactivity clusters is crucial for proper hypothesis generation for uncharacterized small molecules that are subjected to the CPA and requires thorough inspection of the CPA fingerprints of reference compounds.[^2b^, ^9^, ^10^] Thus far, using the CPA we have screened 4,251 reference compounds (e. g., the Library of Pharmacologically Active Compounds (LOPAC), libraries of kinase inhibitors and the Prestwick Chemical Library) and 13,097 small molecules of our in‐house library, which for example includes natural‐product inspired compounds^[11]^ and pseudo‐natural products.^[12]^ For the CPA, human osteosarcoma U2OS cells were treated with compounds for 20 h prior to detection of cellular components and compartments (DNA, RNA, mitochondria, Golgi, endoplasmic reticulum (ER), actin, plasma membrane) using six different dyes. Morphological features are extracted and differences to the control (cells that were treated with DMSO) are expressed as Z scores. The CPA fingerprints used in this study compile Z scores for 579 features. The percentage of significantly altered features (termed induction) is used as a measure of activity and compounds that cause induction >5% are considered active. Fingerprint similarity (termed biosimilarity, BioSim, and based on Pearson correlation) is employed to express similarity between fingerprints and two fingerprints are similar if biosimilarity is >75 %. Investigation of the obtained CPA fingerprints for reference compounds revealed an activity for the DHODH inhibitor brequinar^[13]^ with an induction of 36 % (Figure 1A and 1B). This result is of particular interest as to date DHODH modulation has not been mapped using morphological profiling. Hence, the CPA may be a novel approach to detecting DHODH inhibition in cells. The fingerprint of brequinar is expected to be related to modulation of DHODH if fingerprint similarity to different DHODH inhibitors is observed. Indeed, the CPA revealed high biosimilarity (BioSim >80 %) for brequinar and the structurally unrelated DHODH inhibitor IPP/CNRS‐A017 (see Figure 1A and 1B).^[14]^ Moreover, compound **1** (IPP/CNRS‐A019), which is a much less active derivative of IPP/CNRS‐A017, was not biosimilar to brequinar and IPP/CNRS‐A017 (Figure 1A–1C).^[14]^ Inspecting the reference compounds that share similar fingerprints to brequinar and IPP/CNRS‐A017 revealed biosimilarity to the fingerprint of myxothiazol (Figure 1D and Figure S1). Myxothiazol is an inhibitor of complex III of the mitochondrial electron transport chain.^[15]^ Whereas *de novo* biosynthesis of pyrimidines takes place in the cytosol, DHODH is the only enzyme of the pyrimidine biosynthesis pathway that is localized at the inner mitochondrial membrane.^[16]^ DHODH catalyzes the oxidation of dihydroorotate (DHO) to orotate by transferring electrons from DHO via the cofactor FMN to ubiquinone to yield ubiquinol. Electrons from ubiquinol are transferred further to complex III (cytochrome *b*,*c_1_* complex), thereby recycling ubiquinol that is required for DHODH activity. In this way, pyrimidine biosynthesis is tightly coupled to the activity of complex III, which ensures that pyrimidine nucleotides and, thus, DNA and RNA are synthesized only if cells are supplied with sufficient amount of ATP via the mitochondrial respiratory chain. Alternatively, cells can replenish their pyrimidine pools via the salvage pathway by uptake of circulating pyrimidines.^[5]^ Whereas proliferating cells rely on *de novo* pyrimidine biosynthesis to meet the high demand for nucleotides, the salvage pathway is used by resting and fully differentiated cells.^[5]^

The obtained fingerprints for brequinar, IPP/CNRS‐A017 and myxothiazol are very different from most of the defined CPA bioactivity clusters^[2b]^ as observed in the low dimensional representation of the fingerprints using a UMAP plot (Figure S2A). Brequinar, IPP/CNRS‐A017 and myxothiazol are localized close to the DNA synthesis cluster (Figure S2A and S2B) but most likely form a separate bioactivity cluster. Inhibition of DHODH and, thus, pyrimidine biosynthesis suppresses DNA and RNA synthesis, which also occurs upon impairment of purine biosynthesis, i. e., folate (one carbon) metabolism.^[17]^ Therefore, inhibitors of pyrimidine and purine biosynthesis are expected to display high biosimilarity in the CPA. We recently assigned methotrexate and pralatrexate, which inhibit purine biosynthesis by targeting dihydrofolate reductase (DHFR), to the cluster of DNA synthesis and cell cycle inhibitors.^[9b]^ Interestingly, neither the fingerprint of brequinar nor IPP/CNRS‐A017 were biosimilar to the fingerprint of methotrexate (Figure S3A and S3B) pointing towards a morphological fingerprint that is specific for pyrimidine biosynthesis. Nucleotides are not only required for DNA and RNA synthesis, but are essential metabolites in various processes. Pyrimidines participate in the synthesis of phosphatidylcholine via the generation of the CDP‐choline precursor.^[5]^ Moreover, the synthesis of glucosaminoglycans, *N*‐acetylglucosamine and O‐linked glycosylation depends on the presence of UTP.^[5]^ Therefore, pyrimidines are required for the synthesis of lipids, proteoglycans and protein glycosylation.^[5]^ On the other hand, folate metabolism supplies cells with purines and thymidylate and is essential for remethylation of homocysteine to methionine and production of S‐adenosylmethionine (SAM).^[18]^ Thus, purine biosynthesis is coupled to epigenetic regulation, phospholipid and polyamine synthesis.^[18]^ Most likely, the role of these biosynthetic pathways in processes that are different from nucleic acid synthesis accounts for the dissimilar morphological fingerprints and the lack of biosimilarity in CPA. To explore the cause for this difference in the CPA, we analyzed the biosimilarity of brequinar, IPP/CNRS‐A017 and methotrexate by using features that are related to only one of the used dyes (of note, some stain‐related features may still consider some of the other dyes as well, e. g., correlation of a MitoTracker feature to the one for the ER stain). Whereas such ‘reduced’ fingerprints containing features related to the ER, actin/Golgi/plasma membrane (PM), mitochondrial and RNA staining did not increase fingerprint similarity, considering Hoechst‐related features revealed biosimilarity for these three compounds (Figure S3C). Moreover, increased fingerprint similarity for brequinar and IPP/CNRS‐A017 was detected when features related to DNA, actin/Golgi/PM or RNA were separately compared (Figure S4A) demonstrating that morphological changes related to DNA, actin/Golgi/PM and to lesser extent RNA define the DHODH inhibition fingerprint. In contrast, similarity of two structurally different anti‐folates (trifluridine and pyrimethamine, Figure S4B) to methotrexate was mainly determined by the DNA and ER staining (Figure S4C). These findings demonstrate that pyrimidine and purine biosynthesis share similar morphological changes related to DNA but cause distinct phenotypic alteration of actin/Golgi/PM, RNA or ER, respectively. Thus, the CPA can be employed for differentiating between inhibitors of pyrimidine and purine biosynthesis.

**Figure 2:**
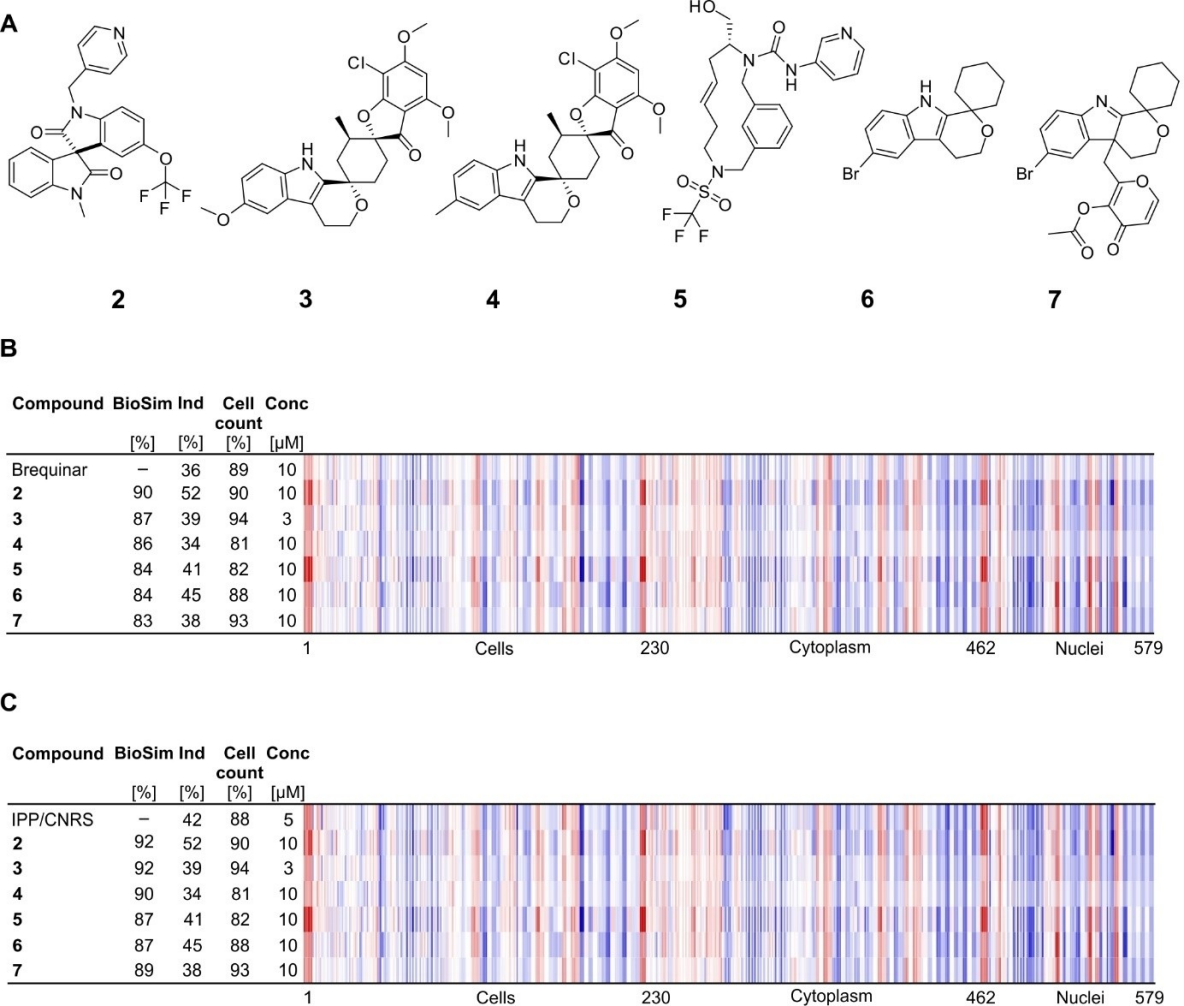
Biosimilarity of in‐house compounds to DHODH inhibitors. (A) Structures of compounds **2**–**7**. (B) Fingerprint comparison of compounds **2**–**7** to brequinar (B) or IPP/CNRS‐A017 (C). The top line fingerprint is set as a reference fingerprint (100 % biological similarity, BioSim) to which the following fingerprints are compared. Values were normalized to the DMSO control. Blue color: decreased feature, red color: increased feature. BioSim: biosimilarity, Ind: induction, Conc: concentration.

We then used the profile of brequinar to search for biosimilar small molecules within our in‐house collection of 13,097 compounds enriched for natural product‐inspired compounds^[11]^ and pseudo‐natural products.^[12]^ Six compounds displayed high similarity to brequinar (Figure 2A and 2B) and an even higher similarity to IPP/CNRS‐A017 (Figure 2C). Compound **2** has a bisindole scaffold. Compounds **3**, and **4** both contain the spiro‐cyclohexyl *4H*‐pyranoindole moiety of the NP fragment **6**,^[19]^ which is also closely related to the indolenine fragment of **7**. Interestingly, we recently reported that the macrocycle **5** ^[20]^ and compound **6** show similar CPA fingerprints to the iron chelator deferoxamine (80 and 81 %, respectively) and to cause accumulation of cells in S phase.^[9b]^ Compounds **5** and **6** displayed higher biosimilarity of 84 % to brequinar and even higher to IPP/CNRS‐A017 (87 %, see Figure 2B and 2 C).

**Figure 3:**
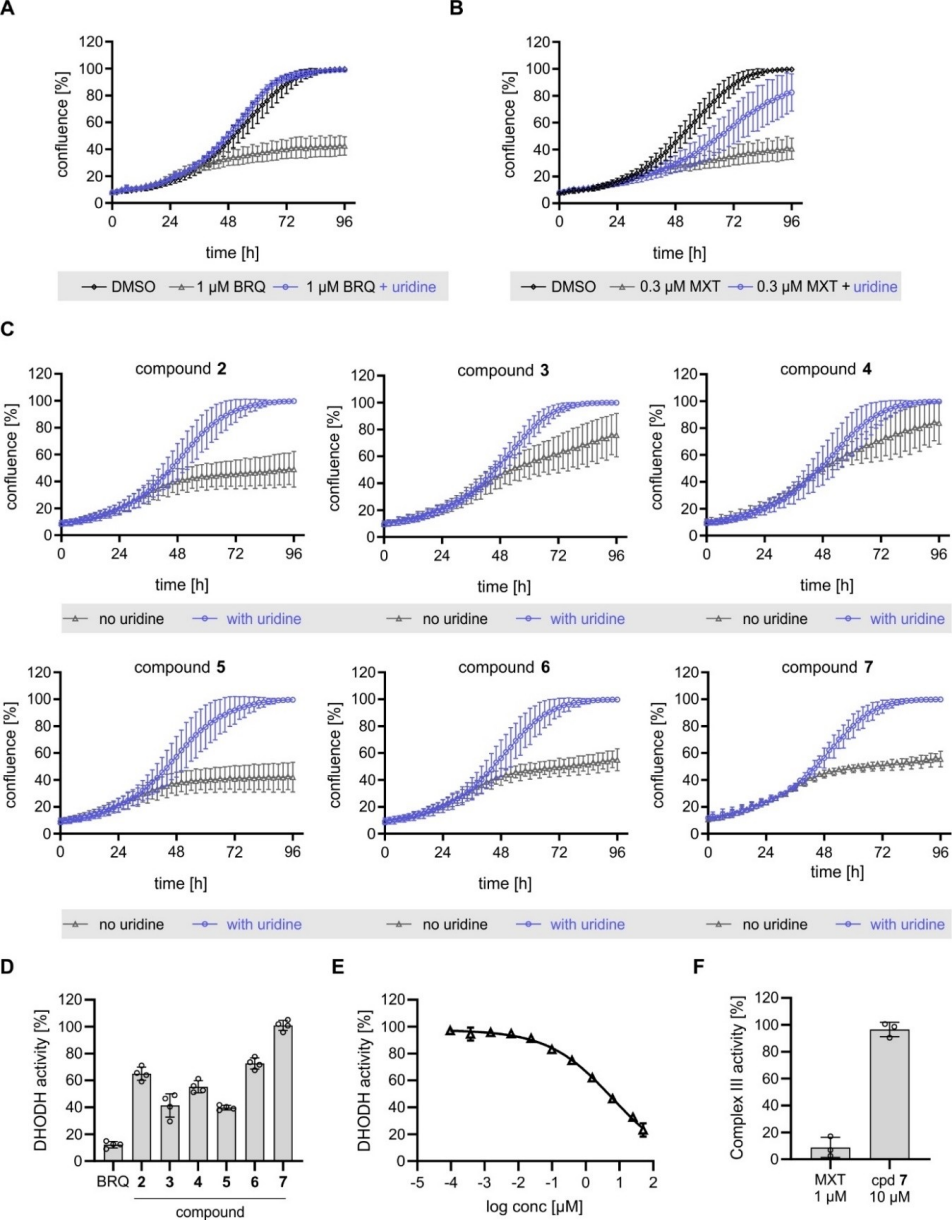
Influence of compounds on cell growth and DHODH activity. (A–C) HCT116 cells were treated with the compounds or DMSO as a control in presence or absence of 100 μM uridine. Cell confluence as a measure of cell growth was monitored over 96 h using IncuCyte ZOOM/S3. (A) Brequinar (BRQ). (B) Myxothiazol (MXT). (C) Compound **2**–**7** (10 μM for **2**, **4**–**7** and 3 μM for **3**). (D–E) *In vitro* DHODH activity. Human DHODH was incubated with compounds **2**–**7** (10 μM), brequinar (1 μM) or DMSO for 30 min prior to initiation of the reaction. (E) Dose‐response curve of compound **5** for inhibition of DHODH. (F) Influence of compound **7** on complex III activity. All data are mean values of three biological replicates (n=3)±SD.

DHODH inhibition suppresses the growth of cells that rely on *de novo* pyrimidine biosynthesis as depletion of pyrimidine nucleotides suppresses DNA synthesis and transcription. Excess of uridine, which can be further converted to UMP and is a source of UTP and CTP, bypasses the need for *de novo* pyrimidine biosynthesis. Supplementation with 100 μM uridine can completely rescue the influence of brequinar on cell growth in U2OS or HCT116 cells (Figure 3A, Figure S5A–5 C and Figure S6, Movies S1–S4). Of note, the growth of HCT116 cells was more sensitive to DHODH inhibition when compared to U2OS cells^[21]^ and, therefore, this cell line was used for further growth rescue experiments.

Myxothiazol also suppressed the growth of HCT116 cells (Figure 3B). However, uridine only partly restored normal cell growth in presence of myxothiazol as opposed to full cell growth recovery by uridine in the presence of brequinar (compare Figure 3A and 3B). A similar influence was observed for the structurally unrelated complex III inhibitor antimycin A (Figure S5D) suggesting that inhibition of complex III itself suppresses cell growth. Thus, full or partial rescue of growth arrest by uridine may differentiate between DHODH and complex III inhibition.

Compounds **2**–**7** inhibited the growth of HCT116 cells to a different extent, which, similar to brequinar, became apparent after 36 h of treatment (Figure 3C). Supplementation of cells with 100 μM uridine completely restored normal cell growth in presence of the compounds (Figure 3C and Figure S7, Movies S5–S8), thus confirming interference with pyrimidine biosynthesis. Moreover, as uridine completely rescued cell growth during treatment with compounds **2**–**7**, these small molecules most likely do not interfere with complex III activity.

We then assessed the influence of the compounds on the *in vitro* enzymatic activity of DHODH. Whereas at 10 μM compound **3** and **5** reduced DHODH activity by more than 50 %, compound **2**, **4** and **6** inhibited DHODH between 27 and 44 % (Figure 3D). Compound **7** was inactive at 10 μM (Figure 3D). Compound **5** dose‐dependently reduced DHODH activity with an IC_50_ of 6.2±3 μM (Figure 3E), whereas the IC_50_ values for the remaining compounds could not be determined due to low solubility at higher concentrations. As myxothiazol and antimycin A do not inhibit *in vitro* DHODH activity (Figure S8) but are biosimilar to brequinar, compounds **7** may target complex III. However, compound **7** did not impair the activity of complex III (Figure 3F).

**Figure 4:**
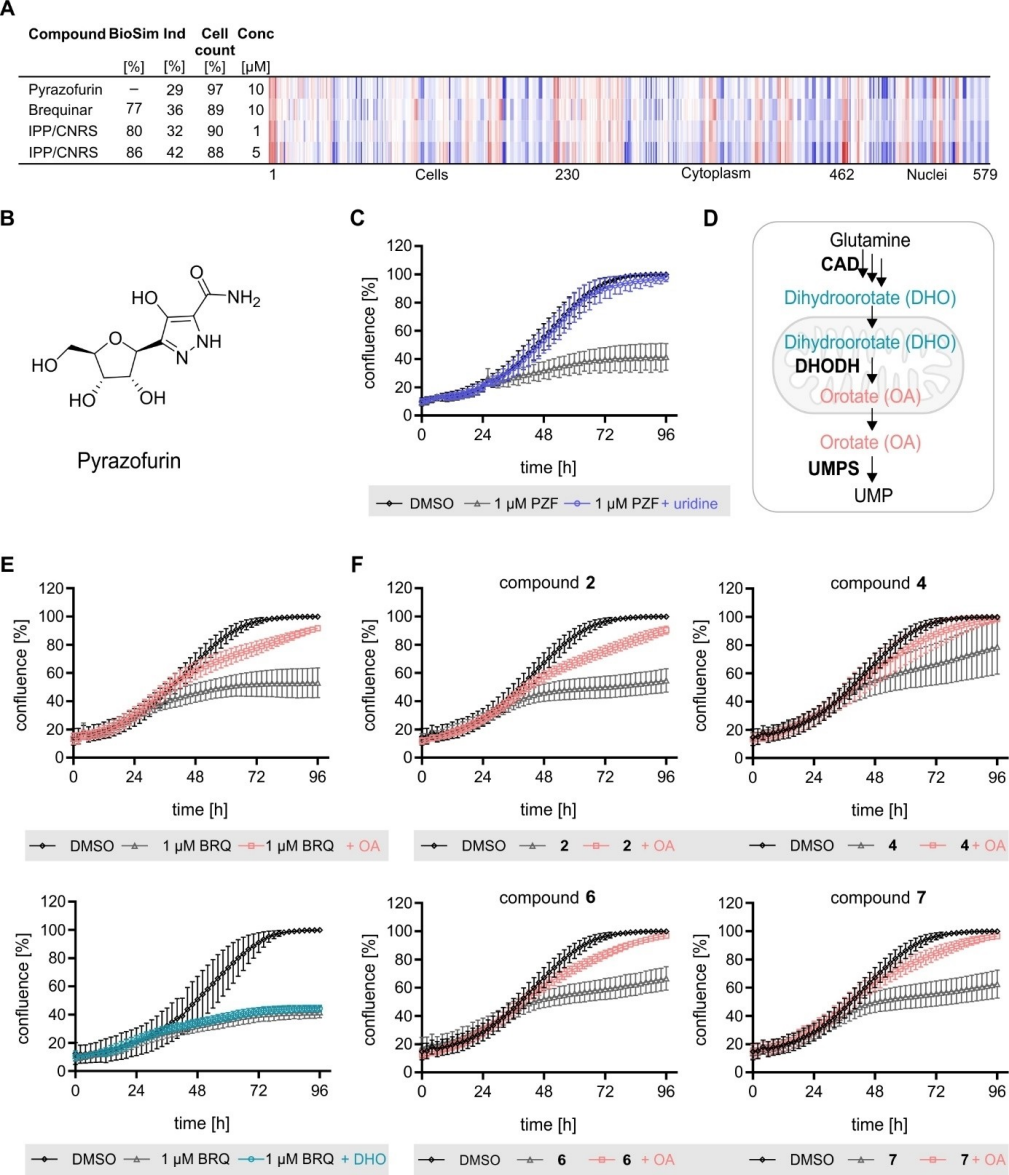
Influence of compounds on cell growth and orotic acid rescue. (A) Fingerprint comparison of pyrazofurin to brequinar and IPP‐CNRS‐A017. The top line fingerprint is set as a reference fingerprint (100 % biological similarity, BioSim) to which the following fingerprints are compared. Values were normalized to the DMSO control. Blue color: decreased feature, red color: increased feature. BioSim: biosimilarity, Ind: induction, Conc: concentration. (B) Structure of pyrazofurin. (C) HCT116 cells were treated with pyrazofurin (PZF) or DMSO as a control in presence or absence of 100 μM uridine. Cell confluence as a measure of cell growth was monitored over 96 h using IncuCyte ZOOM/S3. (D) *De novo* pyrimidine biosynthesis. Glutamine is converted to dihydroorotate (DHO) by CAD (carbamoyl phosphate synthase, aspartate carbamoyltransferase and dihydroorotase). In the mitochondria, DHO is oxidized by DHODH to orotate (OA), which is further converted to UMP by UMPS synthase (UMPS). (E) Influence of DHO or OA on growth suppression induced by brequinar. HCT116 cells were treated with brequinar (BRQ) or DMSO as a control in presence or absence of 1.5 mM DHO or 1.5 mM OA. Cell confluence as a measure of cell growth was monitored over 96 h using IncuCyte ZOOM/S3. (F) Influence of OA on the growth suppression induced by compound **2** (10 μM), **4** (30 μM), **6** (10 μM) and **7** (10 μM). HCT116 cells were treated with the compounds or DMSO as a control in presence or absence of 1.5 mM OA. Cell confluence as a measure of cell growth was monitored over 96 h using IncuCyte ZOOM/S3. All data are mean values (n=3) of three biological replicates±SD.

We recently reported that similar CPA fingerprints can result not only from modulating the same target but also from sharing the same MoA.[^9b^, ^10^] Therefore, we explored whether the DHODH inhibition fingerprint in the CPA is similar to overall inhibition of *de novo* pyrimidine biosynthesis. Besides DHODH, enzymes like CAD (carbamoyl phosphate synthase, aspartate carbamoyltransferase and dihydroorotase) and UMP synthase (UMPS) are involved in *de novo* pyrimidine biosynthesis and their inhibition suppresses this biosynthesis pathway.^[5]^ Interestingly, inhibition of UMPS, which is downstream of DHODH, by the pyrimidine nucleoside analog pyrazofurin^[22]^ yielded a fingerprint with high biosimilarity to brequinar and IPP/CNRS‐A017 (Figure 4A and 4B).

Moreover, pyrazofurin suppressed the growth of HCT116 cells, which was fully rescued by uridine (Figure 4C). These findings demonstrate a common morphological perturbation in the CPA by modulation of *de novo* pyrimidine biosynthesis. Compounds **2**, **4** and **6** only moderately inhibited *in vitro* DHODH activity and compound **7** did not have any influence on the DHODH enzymatic assay. Hence, these compounds may target enzymes of the pyrimidine biosynthesis pathway different from DHODH, e. g., UMPS or CAD, which acts downstream or upstream of DHODH, respectively (Figure 4D). Inhibition of CAD, DHODH and UMPS in cells causes growth suppression that can be counteracted by an excess of uridine or by the product of the respective enzyme. Dihydroorotate (DHO) should rescue inhibition by CAD but not DHODH or UMPS, whereas orotate (OA) should relieve growth suppression cause by CAD or DHODH but not UMPS (Figure 4D). In line with this, 1.5 mM DHO could not restore HCT116 cell growth in presence of brequinar, whereas 1.5 mM OA rescued normal cell proliferation (Figure 4E, Figure S9A–9B and Figure S10–S11). Growth suppression by compounds **2**, **4**, **6** and **7** was also relieved by supplementing cells with OA but not DHO, thus, ruling out modulation of CAD or UMPS and confirming inhibition of DHODH in cells by these small molecules (Figure 4F, Figure S9C and Figure S12–S13).

The identified cluster of compounds that are biosimilar to brequinar shares the inhibition of pyrimidine biosynthesis as a common denominator. However, it unites small molecules addressing different targets like DHODH, UMPS and most likely CAD, which are enzymes that are directly involved in *de novo* pyrimidine biosynthesis. Moreover, inhibitors of mitochondrial complex III activity are also assigned to this cluster due to the tight interplay between DHODH and complex III via the reduction‐oxidation cycle of ubiquinone to ubiquinol.^[16]^ Several biochemical assays would be required to cover these diverse activities, whereas they all can be simultaneously detected using the CPA. The precise target can then easily be identified by exploring cell growth inhibition in presence of metabolites of the pyrimidine biosynthesis like dihydroorotate, orotate or uridine. Whereas CAD inhibition should be rescued by all three metabolites, DHODH inhibition is counteracted by orotate and uridine, and UMPS suppression is rescued by uridine only. Complex III inhibitors like myxothiazol and antimycin A cause growth arrest as well. Interestingly, uridine can only partly rescue growth inhibition in presence of complex III inhibitors. Thus, the rescue pattern can be used to distinguish between modulators of CAD, DHODH and UMPS or complex III. Noteworthy, as CPA fingerprints are obtained after 20 h of compound treatment, the CPA can detect DHODH inhibition earlier than the time that is needed for growth inhibition (48 h for U2OS cells), which may partly be explained by the various processes besides DNA synthesis and cell cycle regulation that depend on the availability of pyrimidines.

The described cluster allows for the rapid identification of small molecules impairing pyrimidine biosynthesis and revealed biosimilarity of brequinar to six in‐house compounds that we validate as DHODH inhibitors. Although they do not potently inhibit the activity of recombinantly expressed N‐terminally truncated DHODH at 10 μM, at this concentration most compounds suppressed cell growth to the same extent as 1 μM brequinar. Therefore, detection of DHODH inhibition in the native environment of the enzyme, i. e., in the mitochondria, appears better suited for the evaluation of compound collections regarding DHODH modulation in cells.

As recently reported, an analysis of CPA fingerprints of macrocycle **5** and compound **6** revealed an increase in the percentage of cells in S phase.^[9b]^ Modulation of DHODH by **5** and **6** is in line with inhibition of DNA synthesis and cell cycle progression. However, the precise mechanism of action of **5** and **6** as DHODH inhibitors was enabled by constantly growing our data set and the inclusion of DHODH inhibitors like brequinar and IPP/CNRS‐A017 as novel reference compounds. Of note, our initial findings pointed towards a similar target for compounds **5** and **6** as detected using hierarchical clustering,^[9b]^ which the current study confirms.

DHODH is a validated target for therapeutic applications and the DHODH inhibitor leflunomide and its active metabolite teriflunomide are approved for the treatment of autoimmune diseases like rheumatoid arthritis and relapsing multiple sclerosis.^[6]^ Modulation of DHODH activity induces differentiation of myeloid cells and reduces the level of leukemia‐initiating cells in a model of acute myeloid leukemia.^[7]^ Moreover, DHODH is considered a therapeutic target in infectious and viral diseases[^8a^, ^23^] including SARS‐CoV‐2.^[8b]^ The CPA can be employed to detect inhibition of pyrimidine biosynthesis in compound collections and promises to uncover further chemotypes that target DHODH, UMPS or CAD that may be starting points for drug discovery research.

## Conclusion

Morphological profiling using the Cell Painting assay enables identification of inhibitors of pyrimidine biosynthesis in an unbiased manner. The CPA can differentiate between the impairment of *de novo* pyrimidine biosynthesis and folate metabolism, which is essential for purine biosynthesis. Depletion of nucleotides and inhibition of DNA synthesis in general is a valid anti‐cancer approach. In particular, DHODH inhibitors are applied in autoimmune disorders and may be beneficial for the treatment of viral diseases including SARS‐CoV‐2. Therefore, the CPA offers an unbiased, easy and fast approach to probe compound collections for depletion of pyrimidine nucleotides that may fuel drug discovery programs aimed at inhibition of pyrimidine biosynthesis.

## Experimental Section

**Materials**: Dulbecco's Modified Eagle's medium (DMEM), L‐glutamine, sodium pyruvate and non‐essential amino acids were obtained from PAN Biotech. Fetal bovine serum (FBS) was purchased from Gibco, Thermo Fisher Scientific Inc., USA. pFN2 A‐hDHODH encoding amino acids aa31–395 of human DHODH was a kind gift from Laboratory of Marco Piccinini, Department of Oncology, University of Turin.^[24]^ L‐dihydroorotic acid (D7128), orotic acid (OA, CatNo O2750) decylubiquonone (D7911), 2,6 dichlorphenolindophenol (D1878), potassium cyanate (60178) and rotenone (R8875) were obtained from Sigma Aldrich, Germany. Uridine (TCI U0020) was obtained from TCI, Japan. Microplates (96‐well, clear, #353072) were obtained from Falcon, USA. MitoTox™ IPP/CNRS‐A017 and IPP/CNRS‐A019 (compound **1**) were provided by the Structural Genomics Consortium. Complex II+III OXPHOS Activity Assay Kit (ab109905) was purchased from Abcam.

**Cell lines**: HCT116 cells (Cat# ACC 581 DSMZ, Germany) and U2OS cells were cultured in DMEM supplemented with 10 % FBS, 2 mM L‐glutamine, 1 mM sodium pyruvate and non‐essential amino acids. Cells were maintained at 37 °C and 5 % CO_2_ in a humidified atmosphere. Cell lines were regularly assayed for mycoplasma contamination and were confirmed to be mycoplasma‐free.

**The cell painting assay**: The Cell Painting assay^[25]^ was performed as described previously.[^9a^, ^26^] U2OS medium (5 μL) was added to each well of a 384‐well plate (PerkinElmer CellCarrier‐384 Ultra) prior to addition of 1,600 U2OS cells per well and incubation for 4 h at 37 °C. Standard stock concentration of compounds was 10 mM. Stock concentrations for very potent compounds ranged from 2 to 0.1 mM. Compounds were added to the cells using the Echo 520 acoustic dispenser (Labcyte) followed by incubation for 20 h at 37 °C. Subsequently, mitochondria were stained with 0.1 μg/μL Mito Tracker Deep Red for 30 min at 37 °C in the dark. Cells were fixed using 3.7 % formaldehyde (in PBS) for 20 min at 37 °C in the dark. Cells were washed three times using PBS before permeabilization using Triton X‐100 for 15 min 37 °C in the dark. After three additional washing steps, 25 μL of a staining solution were added to each well, which contained 1 % BSA, 5 μL/mL Phalloidin (Alexa594 conjugate, Thermo Fisher Scientific, A12381), 25 μg/mL Concanavalin A (Alexa488 conjugate, Thermo Fisher Scientific, Cat. No. C11252), 5 μg/mL Hoechst 33342 (Sigma, Cat. No. B2261‐25 mg), 1.5 μg/mL WGA‐Alexa594 conjugate (Thermo Fisher Scientific, Cat. No. W11262) and 1.5 μM SYTO 14 solution (Thermo Fisher Scientific, Cat. No. S7576). Plates were incubated for 30 min at 37 °C in the dark and washed three times with PBS. Plates were sealed and centrifuged for 1 min at 500 rpm. The plates were prepared in triplicates with shifted layouts to reduce plate effects and imaged using a Micro XL High‐Content Screening System (Molecular Devices) in 5 channels (DAPI: Ex350‐400/Em410‐480; FITC: Ex470‐500/Em510‐540; Spectrum Gold: Ex520‐545/Em560‐585; TxRed: Ex535‐585/Em600‐650; Cy5: Ex605‐650/Em670‐715) with 9 sites per well and 20× magnification (binning 2).

Generated images were processed with the CellProfiler package^[27]^ (https://cellprofiler.org, version 3.0.0) on a computing cluster of the Max Planck Society to extract 1716 cell features. The data was then further aggregated as medians per well (9 sites→1 well), then over the three replicates.

Further analysis was performed with custom Python (https://www.python.org) scripts using the Pandas (https://pandas.pydata.org) and Dask (https://dask.org) data processing libraries as well as the Scientific Python (https://scipy.org) package (separate publication to follow).

A subset of highly reproducible and robust features was determined using the procedure by Woehrmann et al.^[28]^ and as previously described.^[9a]^ A set of robust 579 features that was used for all further analyses. The phenotypic fingerprints were compiled from the Z‐scores of all individual cellular features, where the Z‐score is a measure of how far a data point is from a median value.

Specifically, Z‐scores of test compounds were calculated relative to the Median of DMSO controls. Thus, the Z‐score of a test compound defines how many MADs (Median Absolute Deviations) the measured value is from the Median of the controls as illustrated by the following formula

$$score=\frac{{value}_{meas.}-{Median}_{Controls}}{{MAD}_{Controls}}$$

The phenotypic compound fingerprint is then determined as the list of Z‐scores of all 579 features for one compound.

An induction value was determined as a measure of bioactivity of each compound as the fraction of significantly changed features (in percent):

$$Induction\;\left[\%\right]=\frac{number\;of\;features\;with\;abs.\;values\;>3}{total\;number\;of\;features}$$

Similarities of phenotypic fingerprints (BioSim) were calculated from the correlation distances (CD) between two fingerprints:

$$CD=1-\frac{\left(u-\bar{u}\right)\;\cdot \;\left(v-\bar{v}\right)}{{∥(u-\bar{u})∥}_{2}{∥(v-\bar{v})∥}_{2}}$$

(https://docs.scipy.org/doc/scipy/reference/generated/scipy.spatial.distance.correlation.html), where $\bar{x}$ is the mean of the elements of x , x·y is the dot product of x and y , and ${∥x∥}_{2}$ is the Euclidean norm of x :

$${∥x∥}_{2}=\sqrt{x_{1}^{2}+x_{2}^{2}+\cdots +x_{n}^{2}}\;$$

The BioSim is then defined as:

BioSim=1-CD ; (Values<0 are set to 0)

The term “1−CD” is identical to the Pearson correlation. The BioSim is expressed in percent and values smaller than 0 are set to 0.

The compounds with the most similar fingerprints were determined from a set of 4,217 reference compounds that was also measured in the assay.

**DHODH rescue assay**: 2,000 HCT116 or U2OS cells per well were seeded in a clear 96‐well plate and incubated as described above overnight prior to treatment. The next day, the medium was replaced by fresh compound or DMSO containing medium supplemented with control solvent or uridine (100 μM), OA (1.5 mM) or DHO (1.5 mM). Cell growth was monitored every 2 h for a total of 96 h after treatment start using an IncuCyte S3 (Essen BioScience). The cell confluence was quantified as a measure of cell growth using the IncuCyte S3 software (Essen BioScience).

**Expression and purification of human DHODH**: The N‐truncated form of hDHODH (aa31–395) pFN2a plasmid producing an N‐terminal GST fusion protein was a gift from Marco Piccinini, Department of Oncology, University of Turin.^[24]^ The plasmid pFN2A‐hDHODH encoding amino acids 31–395 of human DHODH was transformed into BL21 (DE3) *E. coli* strain. Cells were grown in LB medium supplemented with 0.1 mM flavin mononucleotide (Sigma Aldrich, F6750) and 100 μg/mL ampicillin (Gerbu, 1046.0050) at 37 °C up to OD_600_ 0.5–0.7. Expression was induced by adding 0.8 mM IPTG (AppliChem, A1008,0025) and temperature was reduced to 28 °C for 6 hours. A cell pellet from 7.5 l expression culture was resuspended in PBS (2.5 mL/g pellet, 50 mM Na_2_HPO_4_, 50 mM NaH_2_PO_4_, 500 mM NaCl), which had been supplemented with 1 mg/g pellet lysozyme (Sigma Aldrich, 62971), 10 mg/g pellet DNAse I (Roche, 10104129001) and 100 μM protease inhibitor (PMSF, Serva, 2395.02) and lysed by sonication using Bandalin Sonoplus HD 2070 (5 min, amplitude 90 %, pulse rate 30s). Triton X‐100 (Serva, 39795.02) was added to the lysate to a final concentration of 1 % followed by an incubation for 30 min on ice before centrifugation at 64,000×g for 45 min at 4 °C. The GST‐fused protein was purified from the bacterial lysate using affinity chromatography on immobilized glutathione‐Sepharose column (GSTrap FT 5 mL, GE Healthcare, 17‐5131‐02) and FPLC. The clarified supernatant was loaded to the column and washed with PBS (1 mL/min) for 2 h, followed by elution with 10 mM L‐glutathione (Sigma Aldrich, G4251) in PBS. The GST tag was not removed for further analysis. Factions containing hDHODH‐GST protein were pooled, concentrated with centrifugal filters (Ultra‐50 K, Merck Millipore, UFC905096) and shock‐frozen for further analysis. Protein concentration was determined by Bradford measurement.

**DHODH enzymatic assay**: Enzymatic activity of DHODH was assessed by monitoring the reduction of 2,6‐dichlorophenolinindophenol (DCPIP), which is coupled to the oxidation of dihydroorotate by DHODH. Using a transparent 96 well plate, 40 μL of purified DHODH (aa31–395, final concentration: 1.25 μg/mL) in assay buffer (50 mM Tris pH 8,0, 150 mM KCl, 0,1 % Triton X‐100) was preincubated with 10 μL of the compounds for 30 min at 37 °C followed by 15 min at room temperature. The reaction was initiated by adding 50 μL mastermix (2 mM L‐dihydroorotic acid, 0.2 mM decylubiquonone, 0.12 mM 2,6 dichlorphenolindophenol (DCPIP) in assay buffer). DCPIP reduction was monitored at λ=600 nm using Tecan Spark plate reader for 20 min. For determination of inhibitory activity and IC_50_ values, the slope of the linear curves over 5 min was calculated, normalized to the DMSO control and further analyzed using GraphPad Prism 9 software.

**MitoTox™ complex II+III OXPHOS activity assay kit**: The assay was performed using the MitoTox™ Complex II+III OXPHOS Activity Assay Kit (ab109905, Abcam) according to the manufacturer's instructions. Compounds or DMSO as a control was mixed with succinate solution (electron donor) and oxidized cytochrome c (electron acceptor) in the presence of complex IV inhibitor potassium cyanate (2 mM, 60168, Sigma Aldrich) and complex I inhibitor Rotenone (12 μM, R8875, Sigma Aldrich). To start the electron transfer reaction, bovine heart mitochondria (0.03 mg/mL) were added to the mixture and conversion of oxidized cytochrome c into the reduced form was monitored for 5 min at 550 nm using the Tecan Spark plate reader.

**Quantification and statistical analysis**: Data were either representative of three independent experiments (n) or expressed as mean±SD. All statistical details of the conducted experiments can be found in the respective figure caption.

## Conflict of interest

The authors declare no conflict of interest.

1

## Supporting information

As a service to our authors and readers, this journal provides supporting information supplied by the authors. Such materials are peer reviewed and may be re‐organized for online delivery, but are not copy‐edited or typeset. Technical support issues arising from supporting information (other than missing files) should be addressed to the authors.

Supporting Information

Supporting Information

Supporting Information

Supporting Information

Supporting Information

Supporting Information

Supporting Information

Supporting Information

Supporting Information

## Data Availability

The code for calculating fingerprint biosimilarity in the Cell Painting assays was published previously^[19]^ and is available at https://github.com/mpimp‐comas/2021_grigalunas_burhop_zinken/blob/master/cpa.py.
