# Supplementary material for: Identification of Dihydroorotate Dehydrogenase Inhibitors Using the Cell Painting Assay
Source: Chembiochem. 2022 Oct 13;23(22):e202200475. doi: 10.1002/cbic.202200475 (PMC9828254; doi:10.1002/cbic.202200475)
Supplement: Supplementary file 1 — Supporting Information [file CBIC-23-0-s004.pdf]

ChemBioChem

Supporting Information

Identification of Dihydroorotate Dehydrogenase Inhibitors Using the Cell Painting Assay

Beate Schölermann, Jana Bonowski, Michael Grigalunas, Annina Burhop, Yusheng Xie, Joseph G. F. Hoock, Jie Liu, Mark Dow, Adam Nelson, Christine Nowak, Axel Pahl, Sonja Sievers, and Slava Ziegler*

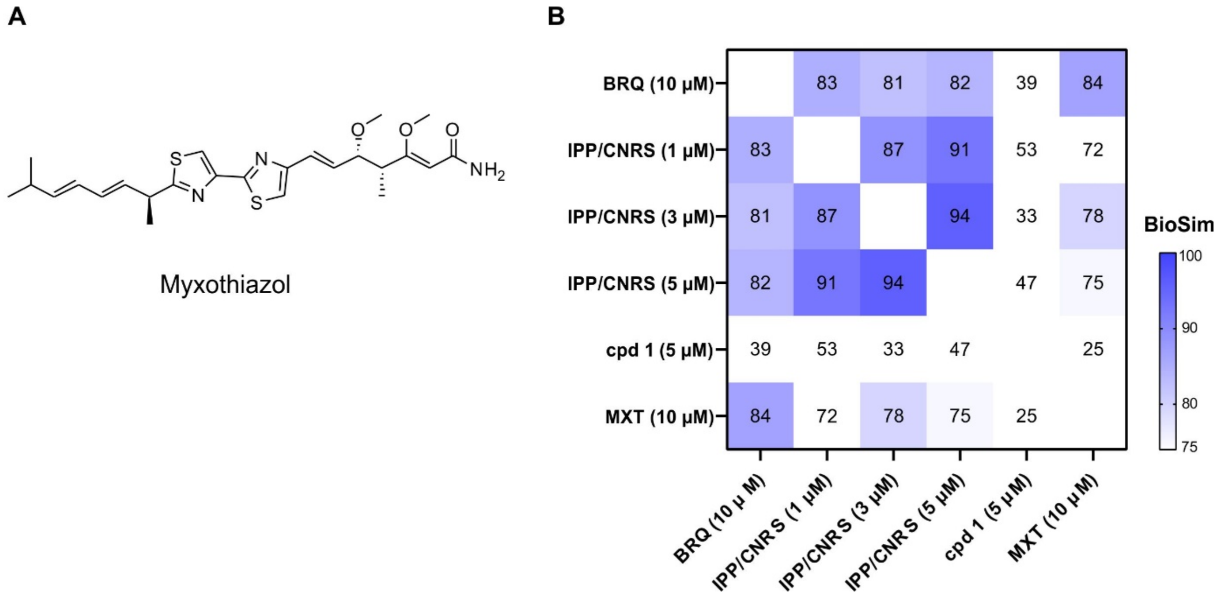

Figure S1 (related to Figure 1). Fingerprint cross-similarity for brequinar, IPP/CNRS-A017, compound 1 and myxothiazol. (A) Structure of myxothiazol. (B) Fingerprint similarities (biosimilarities, BioSim, in %) at the respective concentrations for brequinar (BRQ), IPP/CNRS-A017 (IPP/CNRS), compound **1** (cpd **1**) and myxothiazol (MXT). Fingerprints are considered similar for BioSim > 75 %.

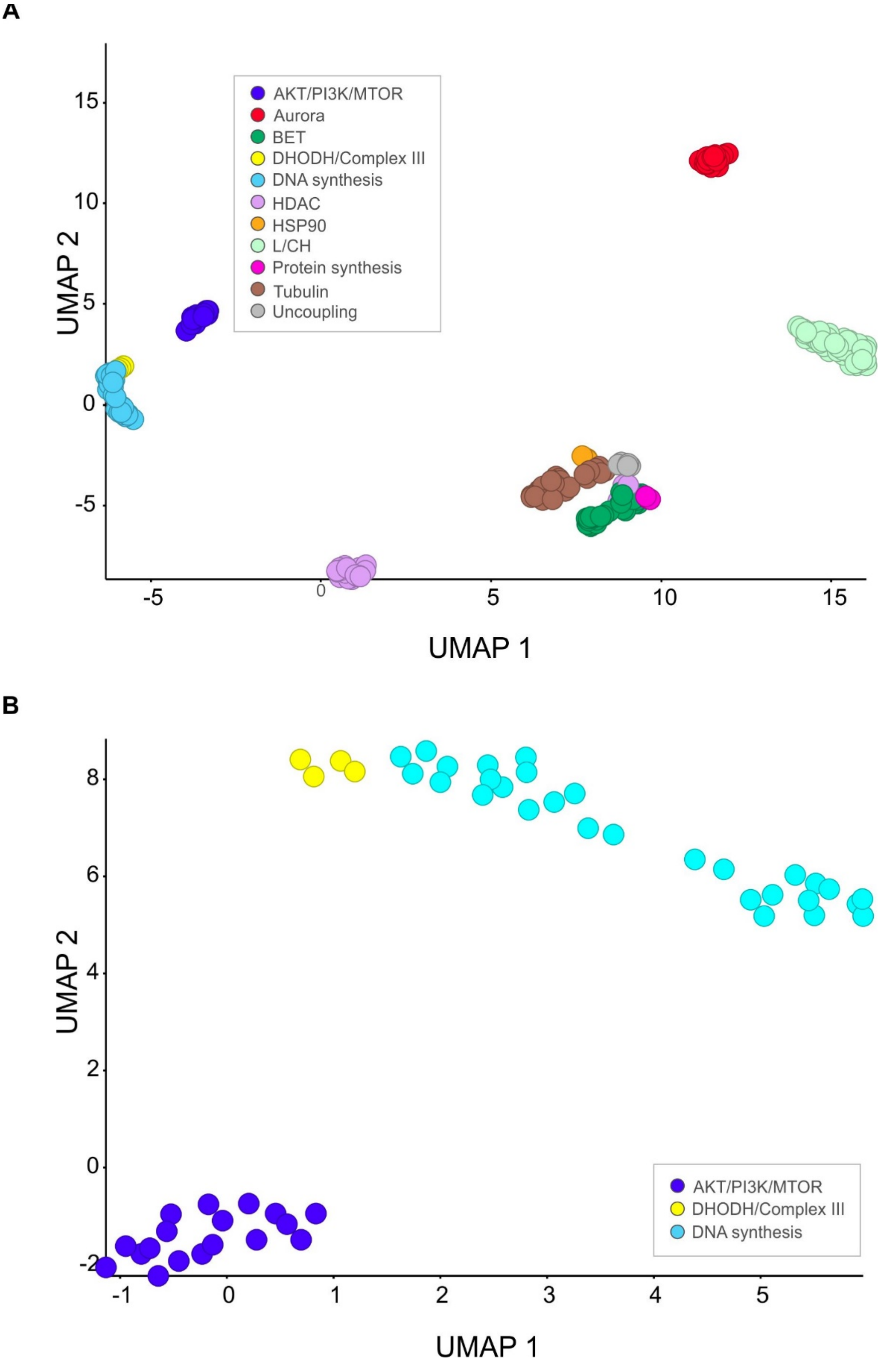

Figure S2 (related to Figure 1). Mapping of inhibitors of DHODH and complex III in the CPA bioactivity cluster space. Brequinar (10 µM), IPP/CNRS-A017 (1 and 5 µM) and myxothiazol (10 µM) were defined as DHODH/Complex III cluster and mapped in the CPA

cluster space thus far comprised of ten different clusters (A). UMAP plot, not normalized, 15 neighbors. (B) UMAP plot (not normalized, 15 neighbors) for the clusters AKT/PI3K/MTOR, DHODH/Complex III and DNA synthesis. L/CH: lysosomotropism/cholesterol homeostasis cluster.

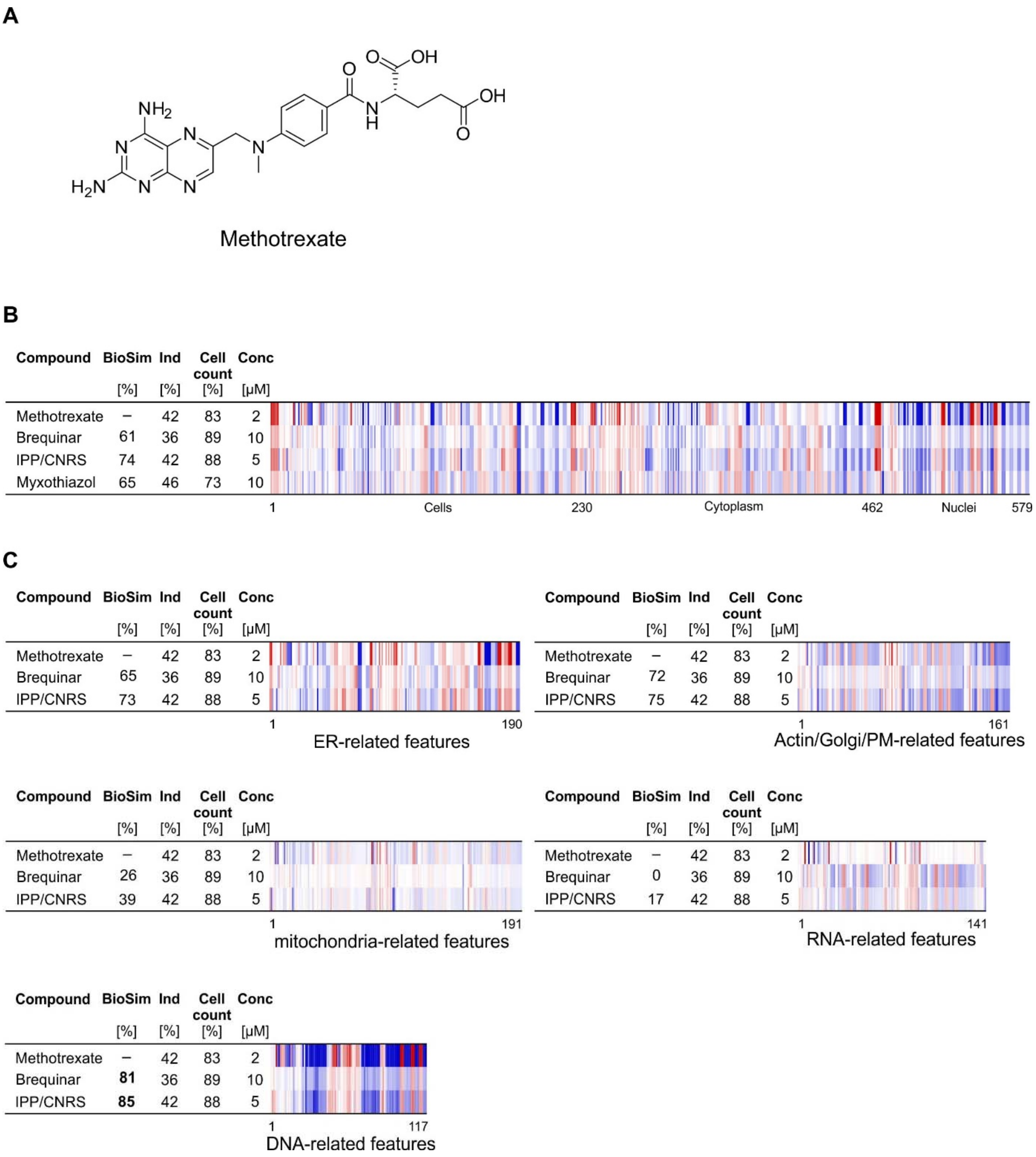

Figure S3 (related to Figure 1). Fingerprint comparison of brequinar, IPP/CNRS-A017 and myxothiazole to methotrexate. (A) Structure of methotrexate. (B) Fingerprint comparison. The top line fingerprint is set as a reference fingerprint (100 % biological similarity, BioSim) to which the following fingerprints are compared. Values were normalized to the DMSO control. Blue color: decreased feature, red color: increased feature. The set of 579

features is divided in features related to the cell (1–229), cytoplasm (230–461) and nuclei (462–579). BioSim: biosimilarity, Ind: induction, Conc: concentration. (C) Fingerprint comparison for methotrexate, brequinar and IPP/CNRS-A017 (IPP/CNRS) considering the features related to each stain only.

A

Compound	BioSim [%]	Ind [%]	Cell count [%]	Conc [µM]
Brequinar	–	36	89	10
IPP/CNRS	81	42	88	5
Myxothiazol	85	46	73	10

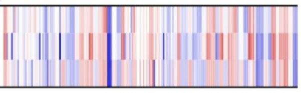

Compound	BioSim [%]	Ind [%]	Cell count [%]	Conc [µM]
Brequinar	–	36	89	10
IPP/CNRS	**90**	42	88	5
Myxothiazol	**86**	46	73	10

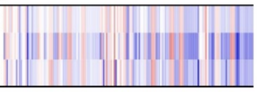

Compound	BioSim [%]	Ind [%]	Cell count [%]	Conc [µM]
Brequinar	–	36	89	10
IPP/CNRS	42	42	88	5
Myxothiazol	48	46	73	10

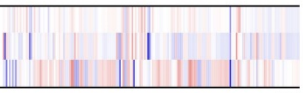

Compound	BioSim [%]	Ind [%]	Cell count [%]	Conc [µM]
Brequinar	–	36	89	10
IPP/CNRS	**86**	42	88	5
Myxothiazol	**93**	46	73	10

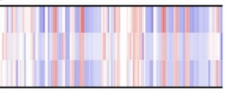

Compound	BioSim [%]	Ind [%]	Cell count [%]	Conc [µM]
Brequinar	–	36	89	10
IPP/CNRS	**94**	42	88	5
Myxothiazol	**95**	46	73	10

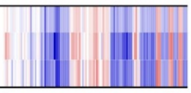

B

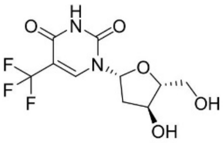

Trifluridine

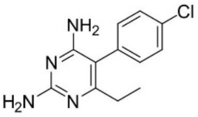

Pyrimethamine

C

Compound	BioSim [%]	Ind [%]	Cell count [%]	Conc [µM]
Methotrexate	–	42	83	2
Trifluridine	93	33	69	10
Pyrimethamine	79	28	95	10

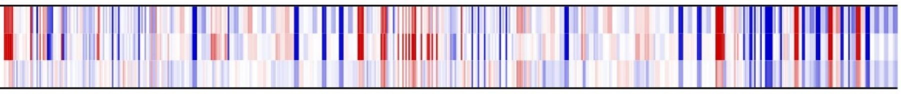

Compound	BioSim [%]	Ind [%]	Cell count [%]	Conc [µM]
Methotrexate	–	42	83	2
Trifluridine	94	33	69	10
Pyrimethamine	74	28	95	10

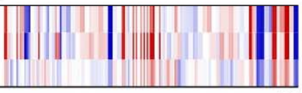

Compound	BioSim [%]	Ind [%]	Cell count [%]	Conc [µM]
Methotrexate	–	42	83	2
Trifluridine	73	33	69	10
Pyrimethamine	45	28	95	10

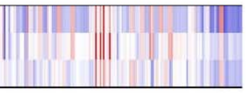

Compound	BioSim [%]	Ind [%]	Cell count [%]	Conc [µM]
Methotrexate	–	42	83	2
Trifluridine	73	33	89	10
Pyrimethamine	54	28	88	10

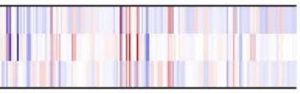

Compound	BioSim [%]	Ind [%]	Cell count [%]	Conc [µM]
Methotrexate	–	42	83	2
Trifluridine	74	33	69	10
Pyrimethamine	53	28	95	10

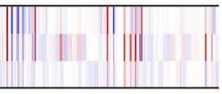

Compound	BioSim [%]	Ind [%]	Cell count [%]	Conc [µM]
Methotrexate	–	42	83	2
Trifluridine	**96**	33	69	10
Pyrimethamine	**91**	28	95	10

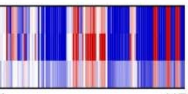

Figure S4 (related to Figure 1). Comparison of fingerprints using separate stain-related features. (A) Comparison for brequinar, IPP/CNRS-A017 (IPP/CNRS) and myxothiazol. (B) Structure of the anti-folates trifluridine and pyrimethamine. (C) Comparison for methotrexate, trifluridine and pyrimethamine. Increase in fingerprint similarity as compared to the full fingerprints is displayed in bold.

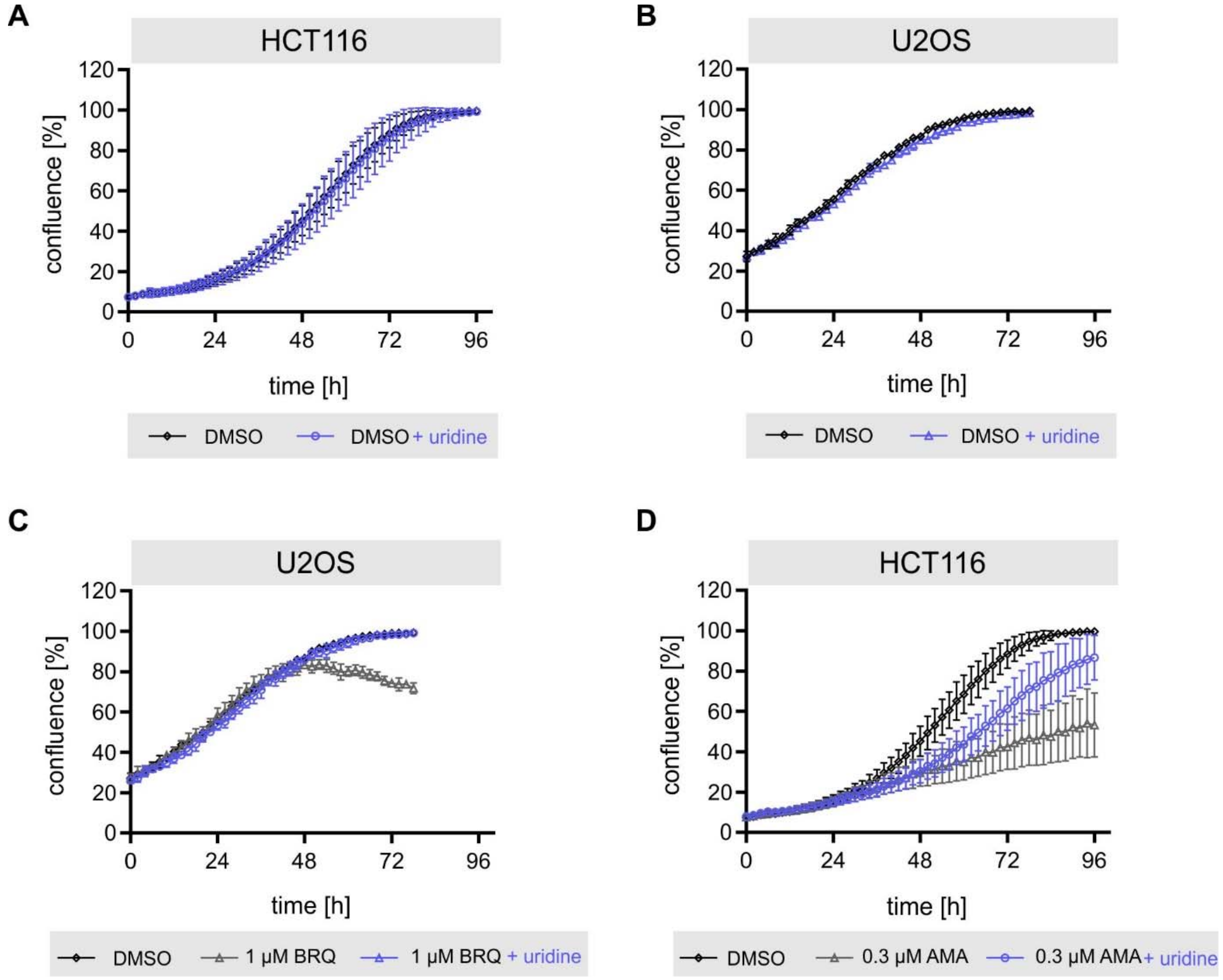

Figure S5 (related to Figure 3). Influence on cell growth in presence or absence of uridine. (A) HCT116 cells were treated with DMSO in presence or absence of 100 µM uridine. (B and C) U2OS cells were treated with DMSO (B) or brequinar (BRQ) (C). (D) HCT116 cells were treated with Antimycin A (AMA). Cell confluence as a measure of cell growth was monitored over 96 h using IncuCyte ZOOM/S3. Data are mean values (n =3) of three biological replicates ± SD (A, and D) or are representative of two biological replicates (B and C).

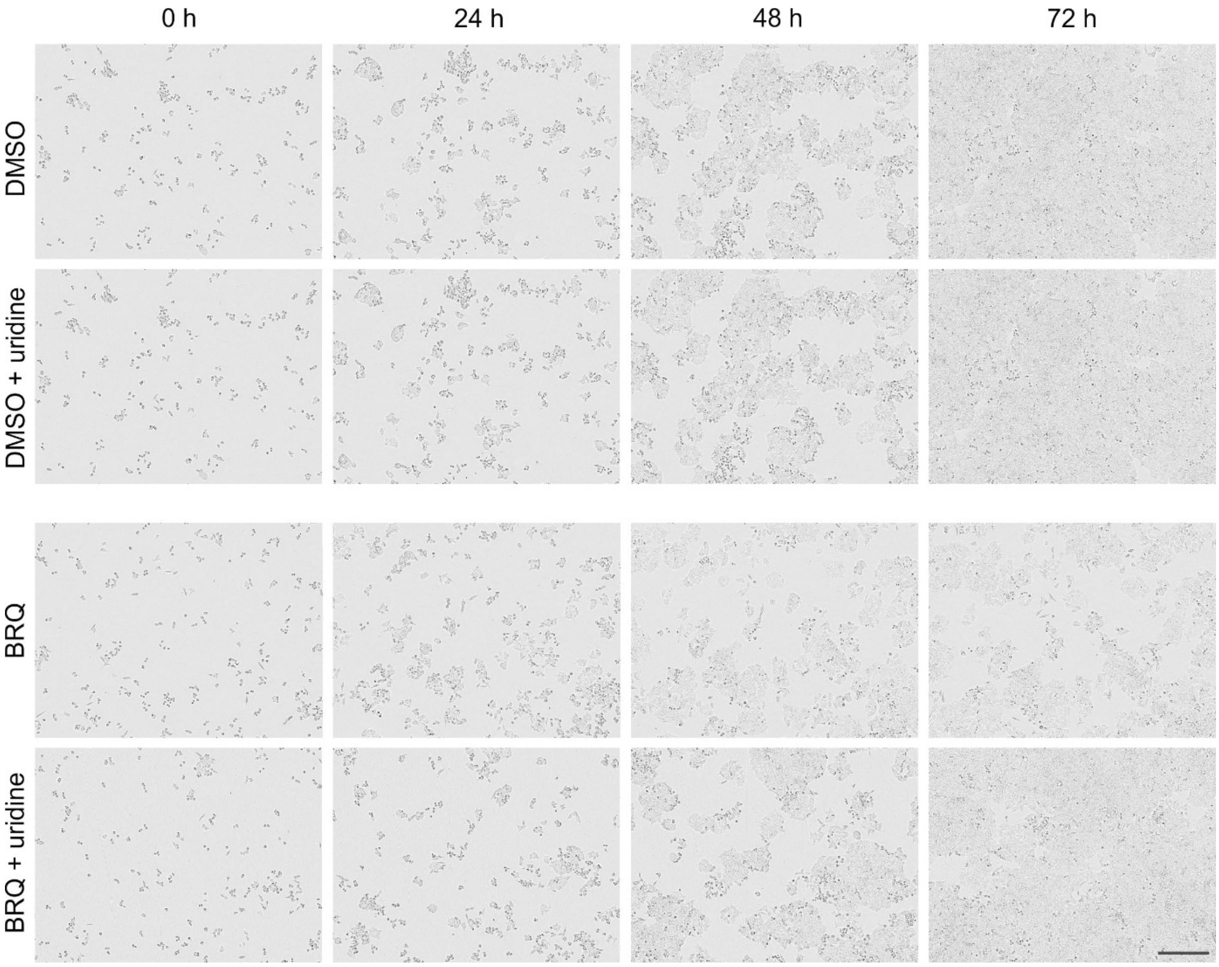

Figure S6 (related to Figure 3). Influence on cell growth. Images of HCT116 cells acquired at different time points upon treatment with DMSO or brequinar (BRQ, 1 µM) in presence or absence of 100 µM uridine. Representative images (for n=3) are shown. Scale bar: 300 µm.

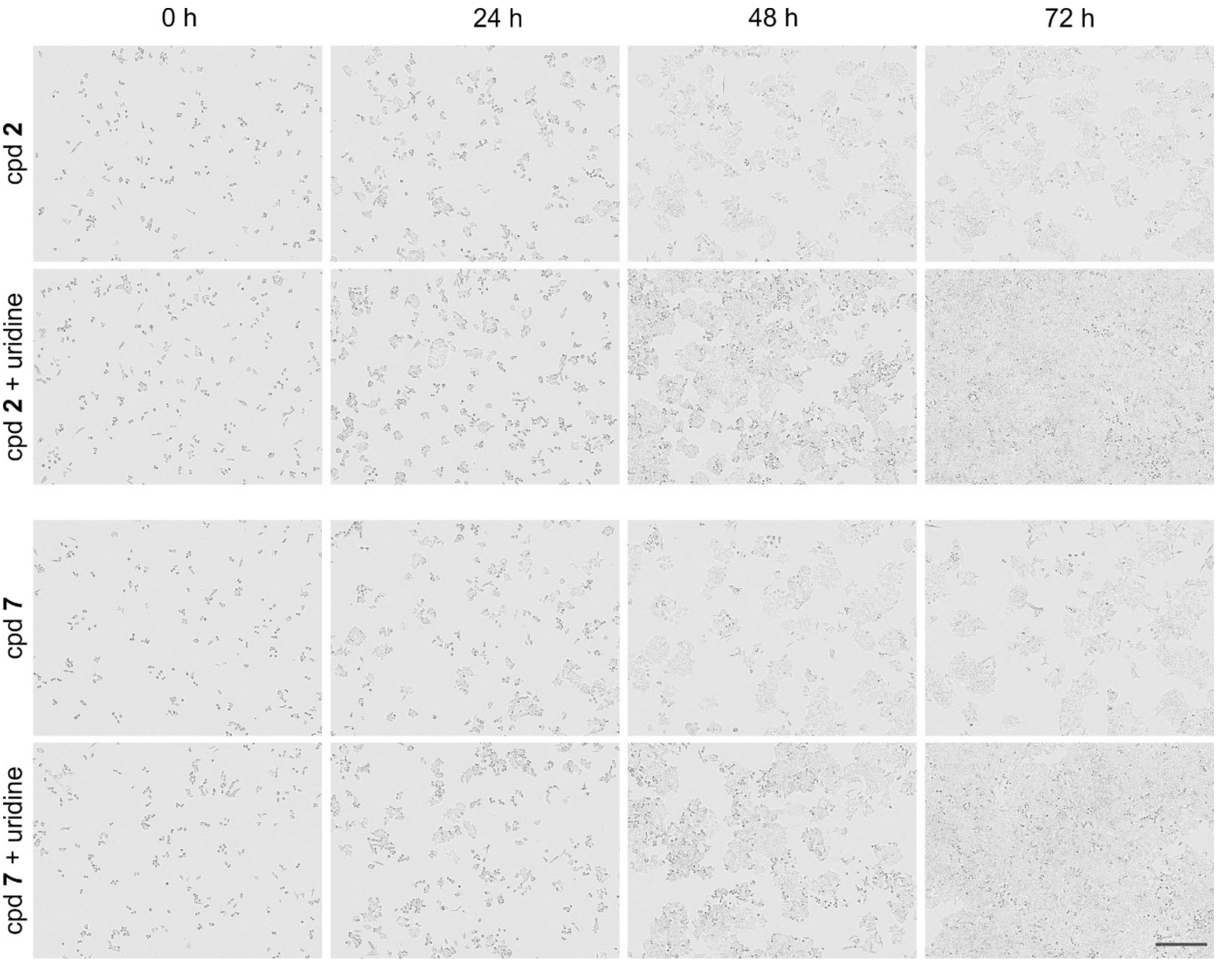

Figure S7 (related to Figure 3). Influence of compound 2 and 7 on cell growth. Images of HCT116 cells acquired at different time points upon treatment with DMSO or compound **2** or **7** (10 μM) in presence or absence of 100 μM uridine. Representative images (of n=3) are shown. Scale bar: 300 μm.

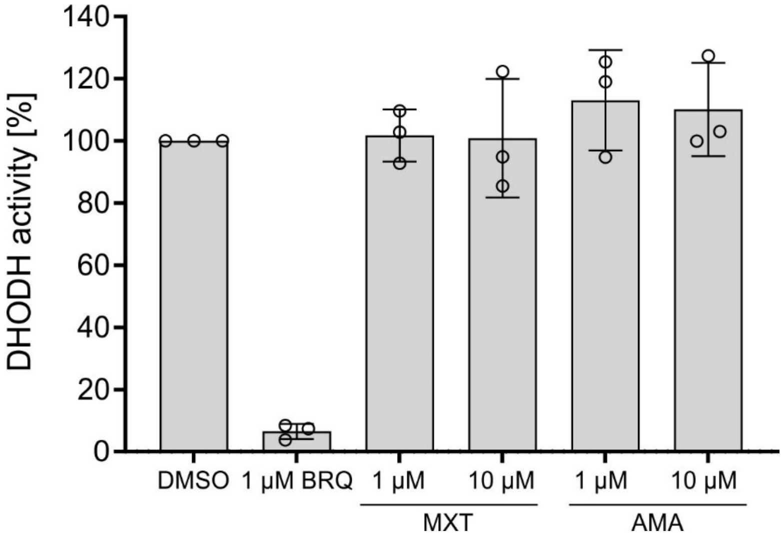

Figure S8 (related to Figure 3): Influence of complex III inhibitors on in vitro DHODH activity. *In vitro* DHODH activity. Human DHODH was incubated with the compounds or DMSO for 30 min prior to initiation of the reaction. All data are mean values of three biological replicates (n =3) ± SD. BRQ: brequinar; MXT: myxothiazol; AMA: antimycin A.

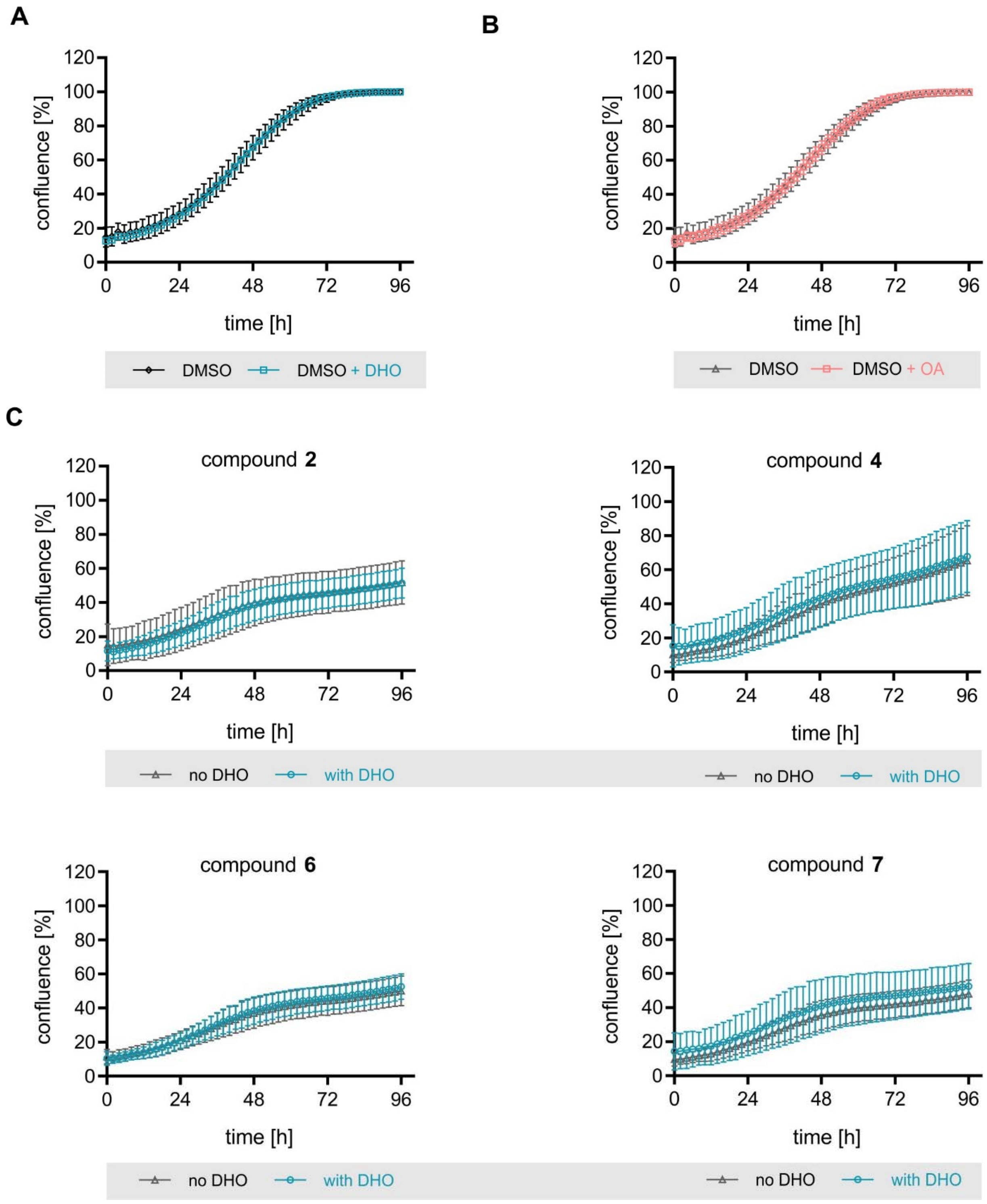

Figure S9 (related to Figure 4). Influence of DHO or OA on cell growth. HCT116 cells were treated with DMSO (A and B) or compound **2** (10 µM), **4** (30 µM), **6** (10 µM) or **7** (10 µM) (C) as a control in presence or absence of 1.5 mM DHO or 1.5 mM OA. Cell confluence

as a measure of cell growth was monitored over 96 h using IncuCyte ZOOM/S3. All data are mean values (n =3) of three biological replicates ± SD.

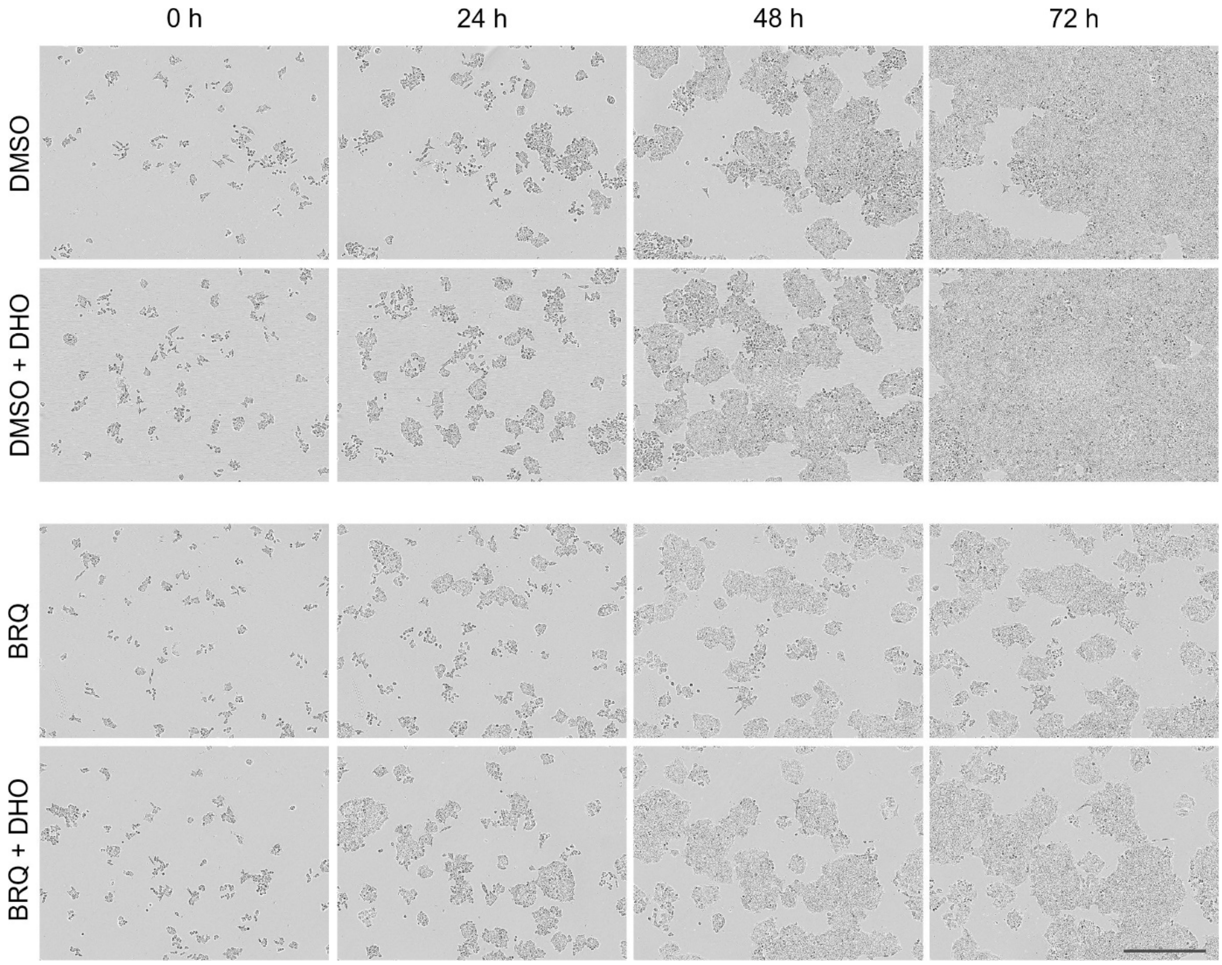

Figure S10 (related to Figure 4). Influence of brequinar on cell growth in presence of dihydroorotate. Images of HCT116 cells acquired at different time points upon treatment with DMSO or brequinar (BRQ, 1 µM) in presence or absence of 1.5 mM dihydroorotate (DHO). Representative images (of n=3) are shown. Scale bar: 500 µm.

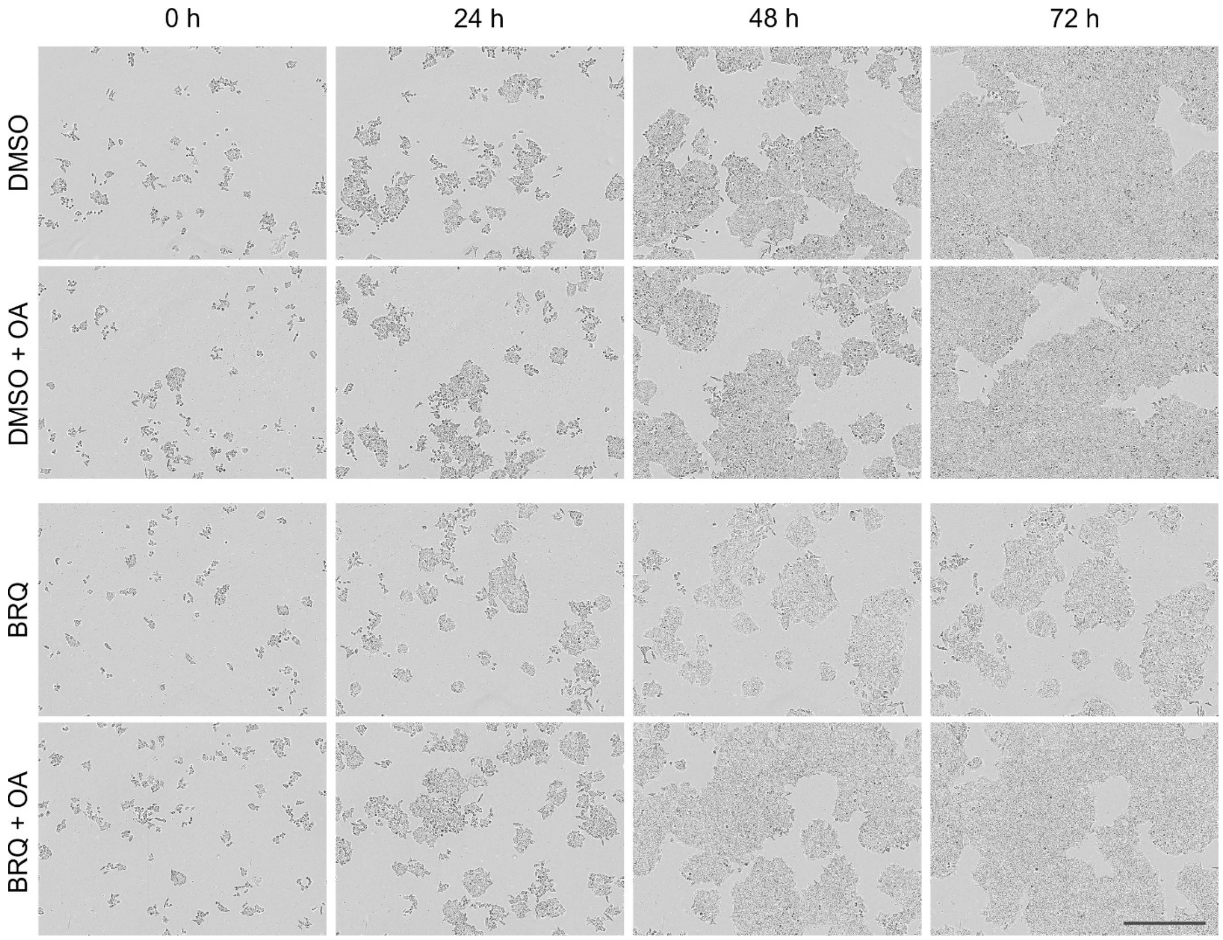

Figure S11 (related to Figure 4). Influence of brequinar on cell growth in presence of orotate. Images of HCT116 cells acquired at different time points upon treatment with DMSO or brequinar (BRQ, 1 μM) in presence or absence of 1.5 mM orotate (OA). Representative images (of n=3) are shown. Scale bar: 500 μm.

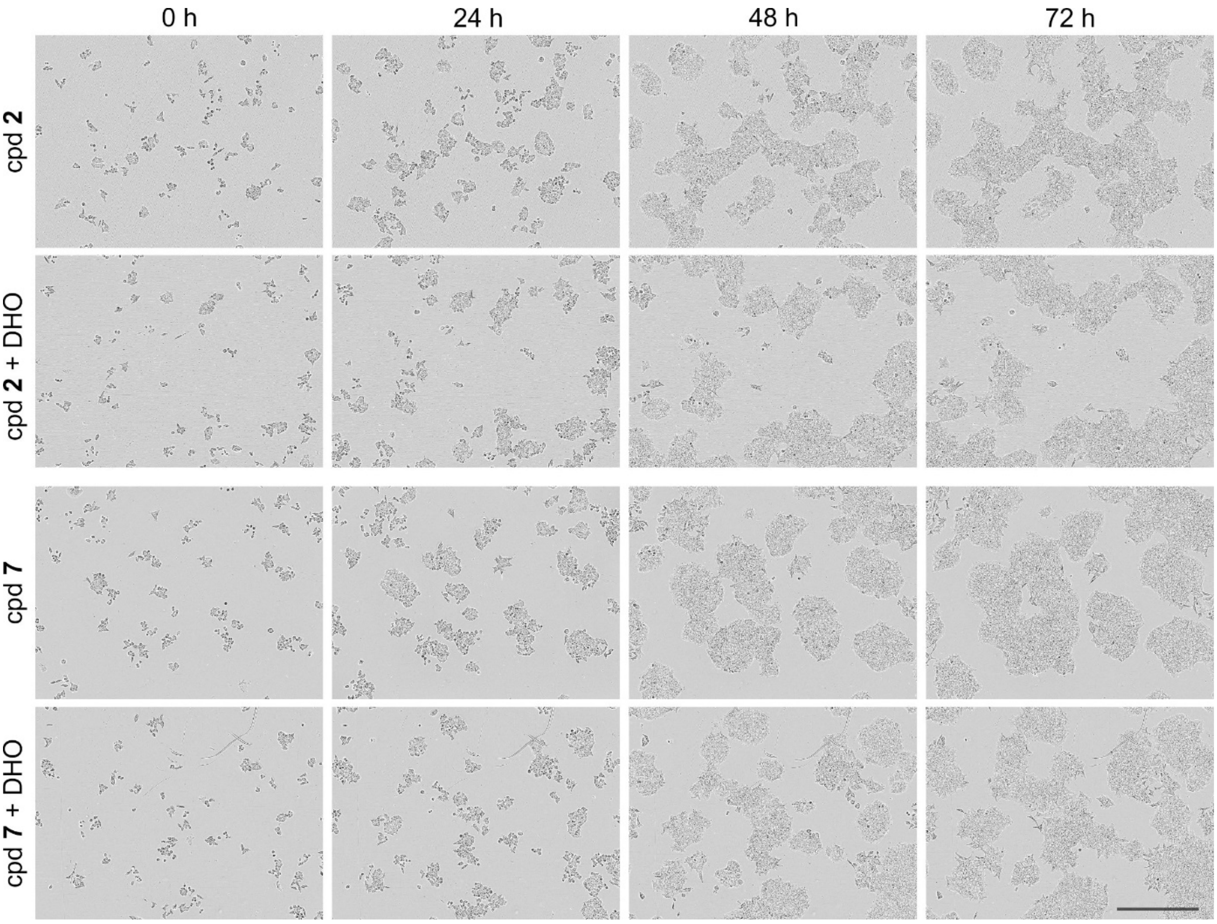

Figure S12 (related to Figure 4). Influence of compound 2 and 7 on cell growth in presence of dihydroorotate. Images of HCT116 cells acquired at different time points upon treatment with DMSO or compound **2** or **7** (10 µM) in presence or absence of 1.5 mM dihydroorotate (DHO). Representative images (of n=3) are shown. Scale bar: 500 µm.

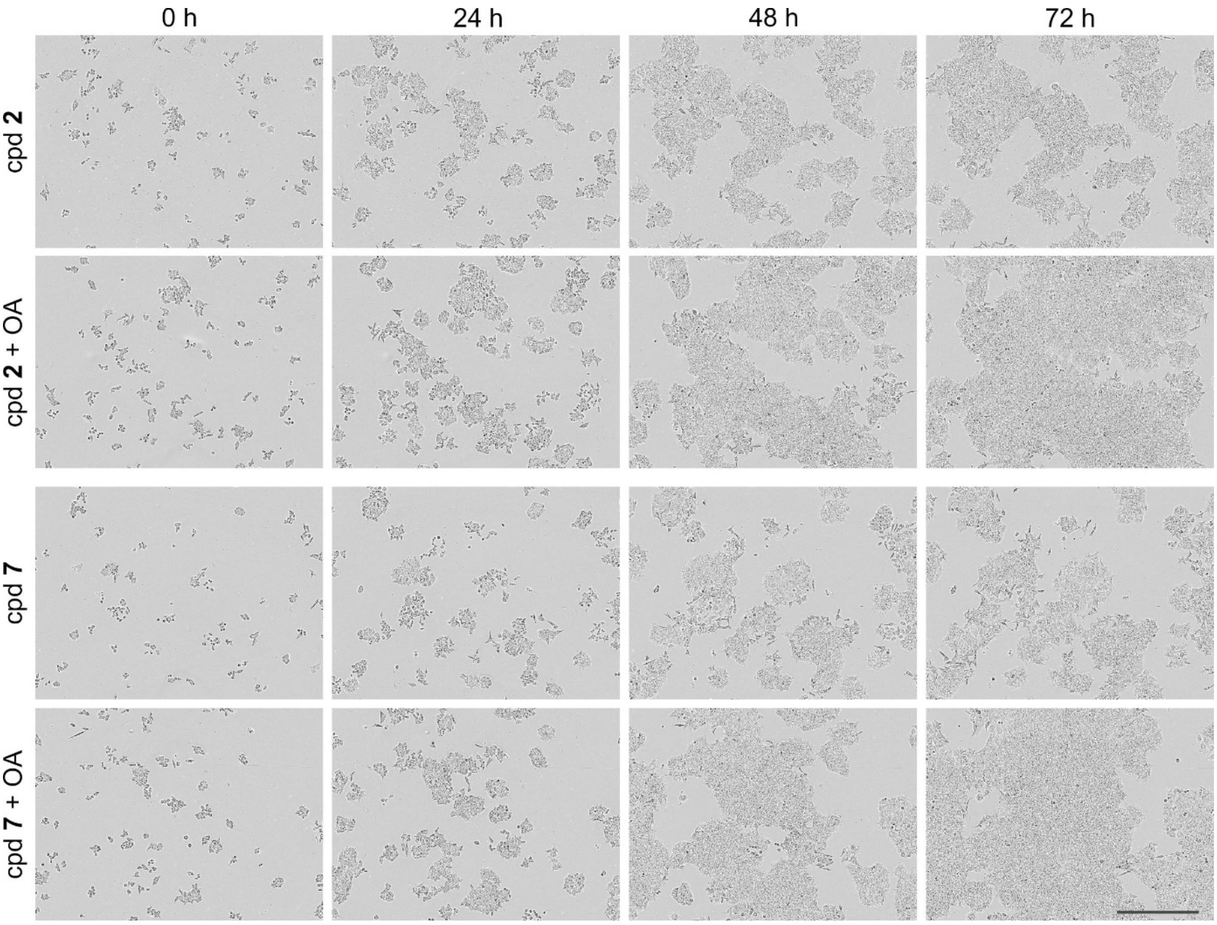

Figure S13 (related to Figure 4). Influence of compound 2 and 7 on cell growth in presence of orotate. Images of HCT116 cells acquired at different time points upon treatment with DMSO or compound **2** or **7** (10 µM) in presence or absence of 1.5 mM orotate (OA). Representative images (of n=3) are shown. Scale bar: 500 µm.

Supporting Movies

Movie S1: HCT116 cells treated with DMSO

Movie S2: HCT116 cells treated with DMSO and 100 μM uridine

Movie S3: HCT116 cells treated with 1 μM brequinar

Movie S4: HCT116 cells treated with 1 μM brequinar and 100 μM uridine

Movie S5: HCT116 cells treated with 10 μM compound **2**

Movie S6: HCT116 cells treated with 10 μM compound **2** and 100 μM uridine

Movie S7: HCT116 cells treated with 10 μM compound **7**

Movie S8: HCT116 cells treated with 10 μM compound **7** and 100 μM uridine

Chemistry

1'-methyl-1-(pyridin-4-ylmethyl)-5-(trifluoromethoxy)-3,3'-spirobi[indoline]-2,2'-dione (2)

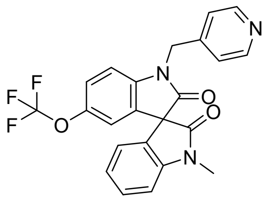

^{1}H NMR (600 MHz, DMSO-*d6*) δ 8.63 – 8.50 (m, 2H), 7.46 (td, *J* = 7.5, 1.8 Hz, 1H), 7.40 – 7.37 (m, 1H), 7.35 (d, *J* = 6.0 Hz, 2H), 7.23 (d, *J* = 7.9 Hz, 1H), 7.18 (d, *J* = 2.5 Hz, 1H), 7.17 (d, *J* = 8.7 Hz, 1H), 7.11 – 7.05 (m, 2H), 5.14 (d, *J* = 17.0 Hz, 1H), 5.04 (d, *J* = 17.0 Hz, 1H), 3.26 (s, 3H). **^{13}C NMR (151 MHz, DMSO-*d6*)** δ 171.9, 170.9, 150.0, 145.4, 144.6, 144.2, 143.0, 130.0, 129.1, 126.6, 123.8, 123.3, 122.9, 121.8, 120.0 (q, *J* = 256.0 Hz), 118.2, 110.8, 109.7, 61.9, 42.4, 27.0. **^{19}F NMR (565 MHz, DMSO-*d6*)** δ -57.3 (s). **HRMS**-ESI (*m/z*): [M + H]$^+$ calculated for $C_{23}H_{17}F_3N_3O_3$ [M+H]$^+$ = 440.1222, found 440.1214.

6'-bromo-4',9'-dihydro-3'H-spiro[cyclohexane-1,1'-pyrano[3,4-b]indole] (6)

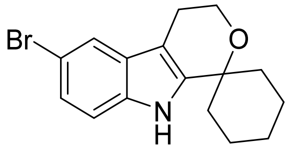

2-(5-Bromo-1H-indol-3-yl)ethan-1-ol (1.0 eq.) was added to an oven-dried microwave vial and dissolved in dry DCM (5 ml). SiO_2·TfOH (5 mol %) and cyclohexanone (1.5 eq.) were added. After flushing of the reaction tube with argon, the reaction mixture was stirred at 22 °C for 2 h. The reaction was stopped by filtration with ethyl acetate (EtOAc). The remaining solvent was evaporated to dryness. Afterwards, the crude reaction mixture was purified by means of silica flash column chromatography (CH/EtOAc 10-35% v/v) to yield **6**.

White Solid (54 mg, 81%); $\mathbf{R_f}$ = 0.37 (CH/EtOAc 10% v/v); **^{1}H NMR** (400 MHz, Chloroform-*d*) δ 7.70 (s, 1H), 7.61 (dt, J = 1.9, 0.6 Hz, 1H), 7.23 (dd, J = 8.6, 1.9 Hz, 1H), 7.18 (dd, J = 8.6, 0.6 Hz, 1H), 3.99 (t, J = 5.5 Hz, 2H), 2.74 (t, J = 5.5 Hz, 2H), 2.04 (m, 2H), 1.77 (dddd, J = 14.9, 7.1, 3.9, 1.9 Hz, 3H), 1.62 (m, 4H), 1.29 (m, 1H) ppm. **HR-MS (ESI)** *m/z* calculated for $C_{16}H_{19}BrNO$ $[M+H]^+$: 320.0645, found 320.0650.

(*S*)-2-((6'-bromo-3',4'-dihydro-4a'H-spiro[cyclohexane-1,1'-pyrano[3,4-b]indol]-4a'-yl)methyl)-4-oxo-4H-pyran-3-yl acetate (7)

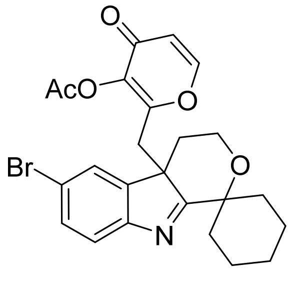

Compound **6** (1.0 eq.) was added to an argon-flushed round-bottom flask and dissolved in dry DCM (1 M). Afterwards, 2-(bromomethyl)-3-((tert-butyldimethylsilyl)oxy)-4H-pyran-4-one (2.0 eq.) was dissolved in TFE (0.32 M) and added to the stirring solution. The reaction mixture was heated to 50 °C and continuously stirred for 30 minutes. After complete conversion of the starting material as indicated by TLC and LC-MS analysis, the solvent was evaporated to

dryness and the crude was directly used in the next reaction (100% conversion of the starting material assumed).

The crude material (1.0 eq.) was dissolved in dry DCM (2 ml) under argon atmosphere. Then, a catalytic amount of DMAP (0.1 eq.) and Et_3N (3.0 eq.) were added and the brown reaction mixture was stirred for 5 minutes at 0 °C. Following, $(Ac)_2O$ (2.4 eq.) was added and the dark brown reaction mixture was continuously stirred at 22 °C. After 30 minutes, the starting material was completely converted into the desired product as indicated by TLC and LC-MS analysis. The reaction was quenched by addition of $NaHCO_3$ (5 ml) and the aqueous layer was subsequently extracted with EtOAc (3 x 5 ml). The combined organic layers were dried under $NaSO_4$ and evaporated to dryness. The resulting crude was purified by gel perfusion chromatography ($CHCl_3$) to isolate compound **7**.

Yellow solid (56 %). **^{1}H NMR** (700 MHz, Chloroform-*d*) δ 7.45 (d, J = 1.5 Hz, 2H), 7.41 (d, J = 5.7 Hz, 1H), 7.29 (s, 1H), 6.27 (d, J = 5.7 Hz, 1H), 4.09 (dt, J = 12.3, 7.7 Hz, 1H), 3.81 (ddd, J = 11.9, 8.2, 3.5 Hz, 1H), 3.50 (d, J = 15.0 Hz, 1H), 3.37 (d, J = 15.0 Hz, 1H), 2.55 (ddd, J = 13.8, 7.4, 3.4 Hz, 1H), 2.36 (s, 3H), 2.18 (d, J = 12.9 Hz, 1H), 2.01 (td, J = 13.3, 12.5, 4.0 Hz, 1H), 1.96 (d, J = 12.0 Hz, 1H), 1.87 (td, J = 13.1, 4.0 Hz, 1H), 1.79 – 1.73 (m, 2H), 1.70 – 1.66 (m, 1H), 1.66 – 1.62 (m, 1H), 1.62 – 1.54 (m, 2H), 1.40 (ddd, J = 16.1, 8.1, 3.8 Hz, 1H) ppm. **^{13}C NMR** (176 MHz, Chloroform-*d*) δ 190.4, 171.3, 167.1, 157.1, 153.6, 143.3, 139.4, 131.8, 125.9, 121.9, 119.5, 116.9, 77.9, 56.0, 55.6, 34.3, 33.5, 33.3, 33.2, 25.2, 21.2, 21.0, 20.4 ppm. **HRMS**-ESI (*m/z*): $[M + H]^+$ calculated for $C_{24}H_{25}BrNO_5$ $[M+H]^+$ = 486.0911, found 486.0910.

NMR spectra

^{1}H NMR Spectrum of **2** (600 MHz, DMSO-*d6*)

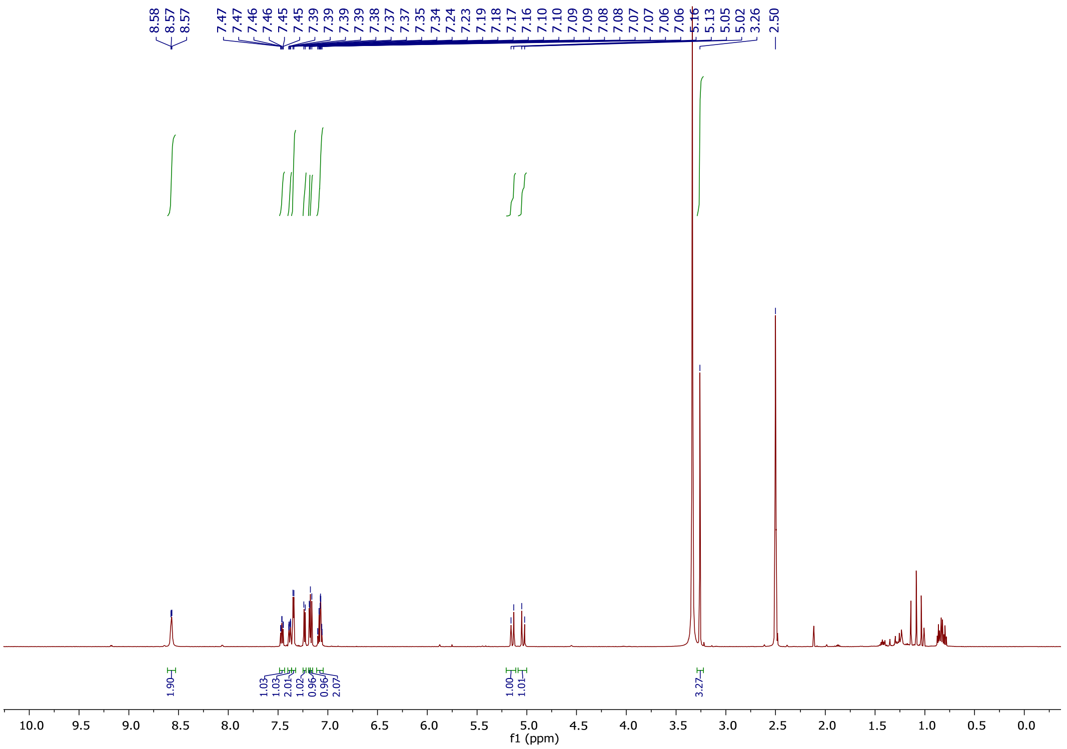

^{13}C NMR Spectrum of **2** (151 MHz, DMSO-*d6*)

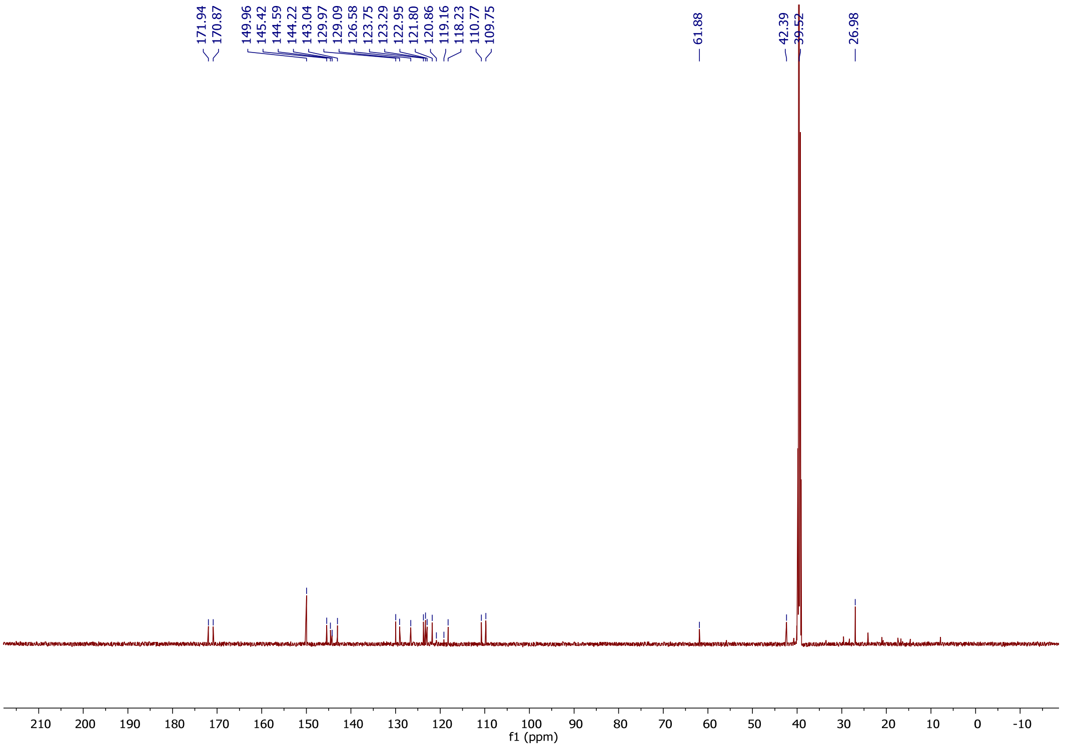

^{19}F NMR Spectrum of **2** (565 MHz, DMSO-*d6*)

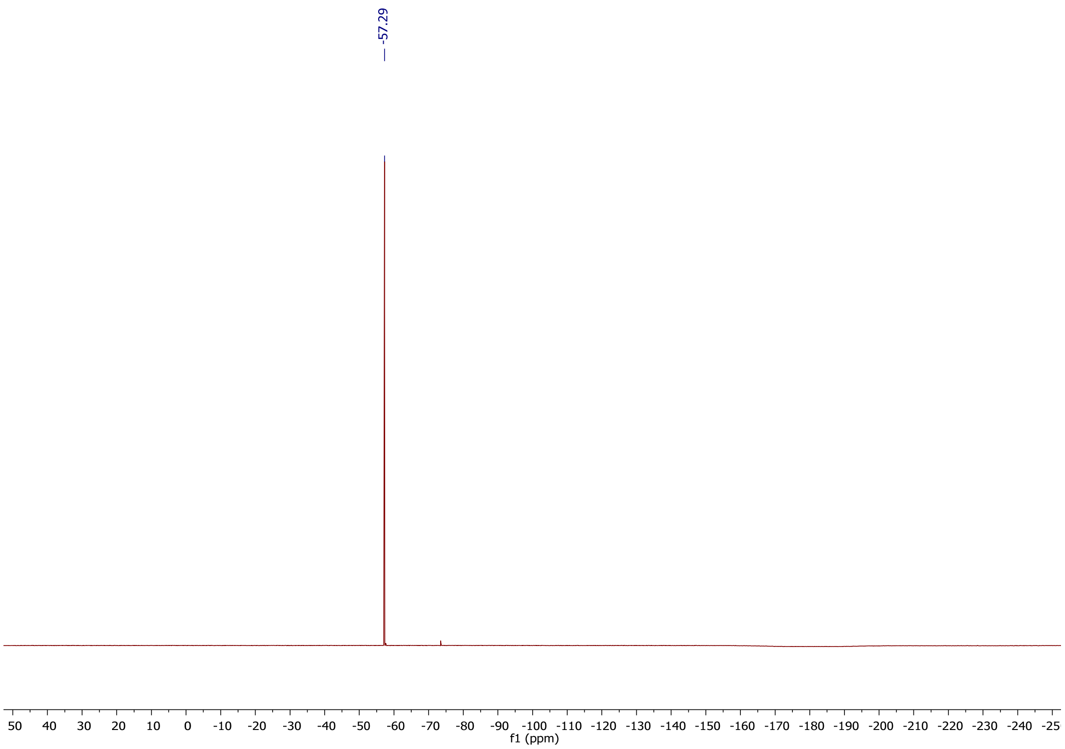

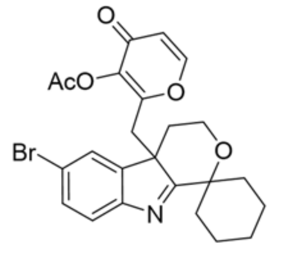
O
AcO
O
Br
O
N

7

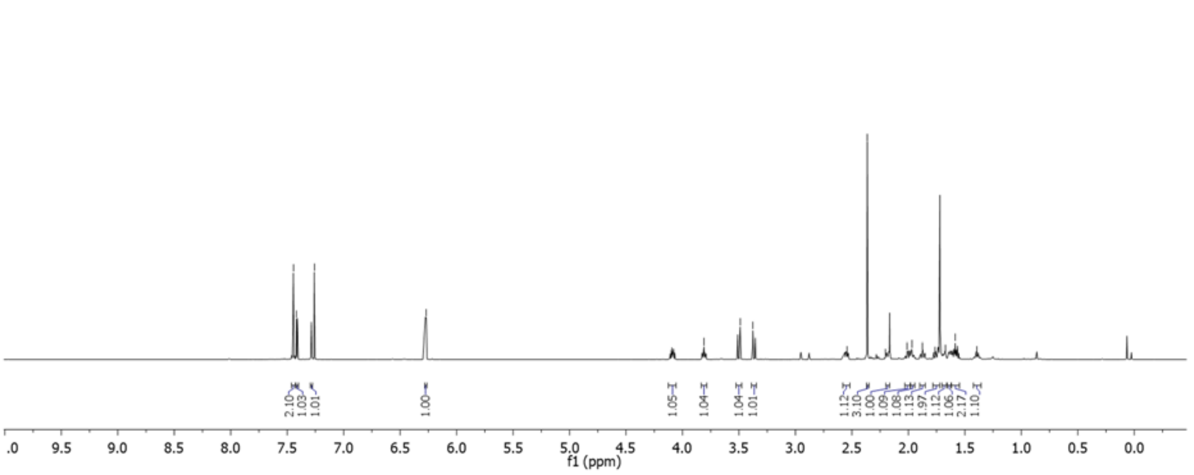
7.45
7.42
7.26
6.27
4.09
3.81
3.49
3.38
2.55
2.36
2.20
2.01
1.97
1.88
1.76
1.67
1.59
1.39
2.10
1.03
1.01
1.00
1.05
1.04
1.04
1.01
1.12
3.10
1.00
1.09
1.08
1.13
1.97
1.12
1.06
2.17
1.10
f1 (ppm)

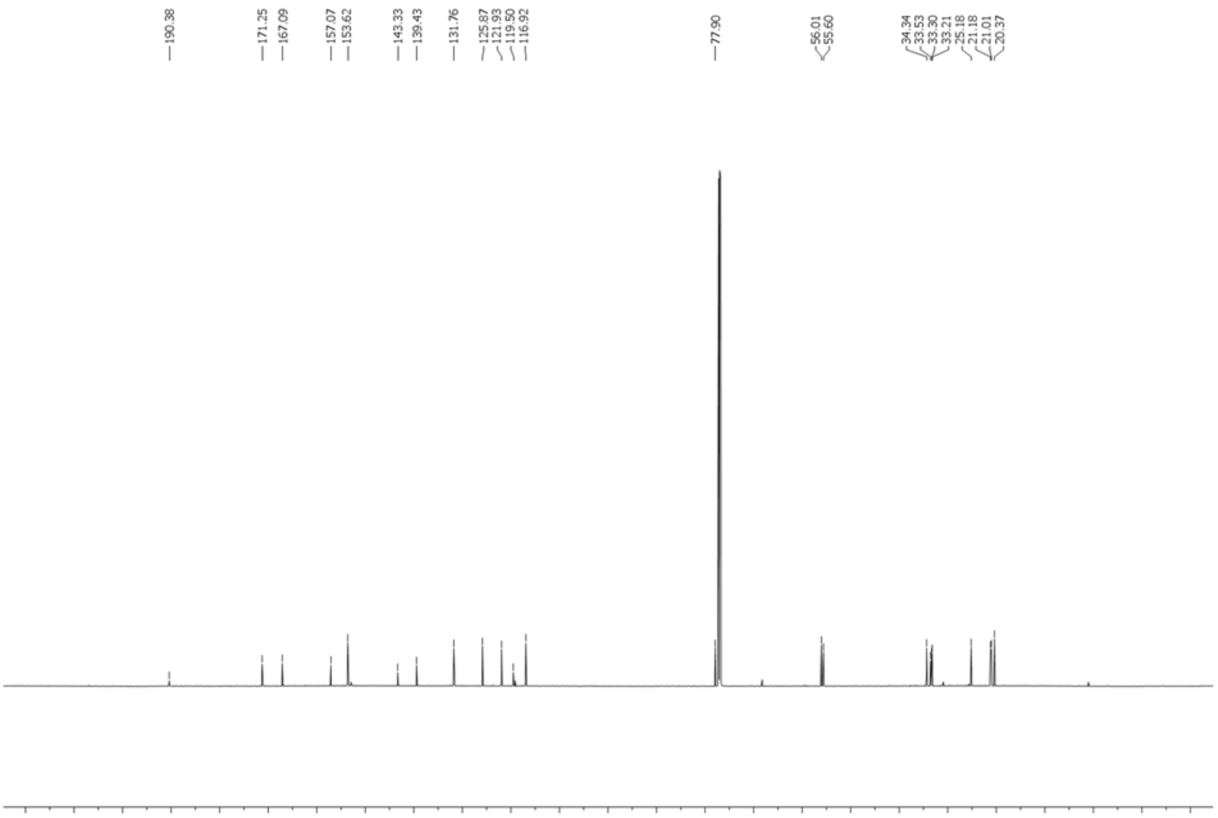
190.38
171.25
167.09
157.07
153.62
143.33
139.43
131.76
125.87
121.93
119.50
116.92
77.90
56.01
55.60
34.34
33.53
33.30
33.21
25.18
21.01
20.37
f1 (ppm)
